# Isolation, Characterization, and Expression Analysis of NAC Transcription Factor from *Andrographis paniculata* (Burm. f.) Nees and Their Role in Andrographolide Production

**DOI:** 10.3390/genes15040422

**Published:** 2024-03-28

**Authors:** Ramesh Kumar, Chavlesh Kumar, Debjani Roy Choudhury, Aashish Ranjan, Ritesh Kumar Raipuria, Kaushik Kumar Dhar Dubey, Ayushi Mishra, Chetan Kumar, Malik Muzafar Manzoor, Ashok Kumar, Abha Kumari, Kuldeep Singh, Gyanendra Pratap Singh, Rakesh Singh

**Affiliations:** 1 Division of Genomic Resources, ICAR-National Bureau of Plant Genetic Resources, New Delhi 110012, Delhi, India; ramesh.manglesha@gmail.com (R.K.); roydebj@gmail.com (D.R.C.); 2 Amity Institute of Biotechnology, Amity University, Noida 201313, Uttar Pradesh, India; dubey.kaushik01@gmail.com (K.K.D.D.); akumari@amity.edu (A.K.); 3 Division of Fruits and Horticultural Technology, ICAR-Indian Agricultural Research Institute, New Delhi 110012, Delhi, India; ckfruits2016@gmail.com; 4 National Institute of Plant Genome Research, Aruna Asaf Ali Marg, New Delhi 110067, Delhi, India; aranjan@nipgr.ac.in (A.R.); raipuriaritesh@gmail.com (R.K.R.); 5 School of Biotechnology, Jawaharlal Nehru University, New Delhi 110067, Delhi, India; ayushi.mishra107@gmail.com; 6 CSIR-Indian Institute of Integrative Medicine, Jammu 180001, Jammu and Kashmir, India; kumarbelwal@gmail.com (C.K.); muzafarmanzoor@gmail.com (M.M.M.); 7 School of Pharmaceutical & Populations Health Informatics, DIP University Mussoorie-Dehradun, Dehradun 248009, Uttrakhand, India; 8 Division of Germplasm Evaluation, ICAR-National Bureau of Plant Genetic Resources, New Delhi 110012, Delhi, India; ashok.kumar28@icar.gov.in; 9 ICAR-National Bureau of Plant Genetic Resources, New Delhi 110012, Delhi, India; kuldeep.singh@icrisat.org (K.S.); gp.singh@icar.gov.in (G.P.S.); 10 International Crops Research Institute for Semi-Arid Tropics, Hyderabad 502324, Telangana, India

**Keywords:** transcription factor, andrographolide, abscisic acid, medicinal plant, phylogenetic analysis, methyl jasmonate, transient expression

## Abstract

*Andrographis paniculata* (Burm. f.) Nees is an important medicinal plant known for its bioactive compound andrographolide. NAC transcription factors (NAM, ATAF1/2, and CUC2) play a crucial role in secondary metabolite production, stress responses, and plant development through hormonal signaling. In this study, a putative partial transcript of three NAC family genes (*ApNAC83*, *ApNAC21 22* and *ApNAC02*) was used to isolate full length genes using RACE. Bioinformatics analyses such as protein structure prediction, cis-acting regulatory elements, and gene ontology analysis were performed. Based on in silico predictions, the diterpenoid profiling of the plant’s leaves (five-week-old) and the real-time PCR-based expression analysis of isolated NAC genes under abscisic acid (ABA) treatment were performed. Additionally, the expression analysis of isolated NAC genes under MeJA treatment and transient expression in *Nicotiana tabacum* was performed. Full-length sequences of three members of the NAC transcription factor family, *ApNAC83* (1102 bp), *ApNAC21 22* (996 bp), and *ApNAC02* (1011 bp), were isolated and subjected to the promoter and gene ontology analysis, which indicated their role in transcriptional regulation, DNA binding, ABA-activated signaling, and stress management. It was observed that ABA treatment leads to a higher accumulation of andrographolide and 14-deoxyandrographolide content, along with the upregulation of *ApNAC02* (9.6-fold) and the downregulation of *ApNAC83* and *ApNAC21 22* in the leaves. With methyl jasmonate treatment, *ApNAC21 22* expression decreased, while *ApNAC02* increased (1.9-fold), with no significant change being observed in *ApNAC83*. The transient expression of the isolated *NAC* genes in a heterologous system (*Nicotiana benthamiana*) demonstrated their functional transcriptional activity, leading to the upregulation of the *NtHMGR* gene, which is related to the terpene pathway in tobacco. The expression analysis and heterologous expression of *ApNAC21 22* and *ApNAC02* indicated their role in andrographolide biosynthesis.

## 1. Introduction

*Andrographis paniculata* (Burm. f.) Nees is one of the most important medicinal plant species from the family Acanthaceae [1]. Due to its extreme bitterness, it is also referred to as the “King of bitter” [2]. *A. paniculata* is naturally distributed and commercially cultivated in countries like India, China, Mauritius, and Thailand [3]. In Ayurveda, Unani, and traditional Chinese medicines, *A. paniculata* is widely used as a herb [4]. It contains several essential bioactive compounds like andrographolide, neo andrographolide, and 14-deoxy-11,12-didehydroandrographolide, of which andrographolide is a principal bioactive compound and is mainly accumulated in leaves [5]. Andrographolide is a diterpenoid class of secondary metabolites that mainly accumulates in the leaves of the plant and is synthesized by the mevalonate (MVA) and methylerythritol 4-phosphate (MEP) pathway [5]. The various important pharmacological effects, such as immunostimulatory, anticancer, anti-hepatitis, cardiovascular, anti-HIV, anti-inflammatory, anti-microbial, antimalarial, cytotoxic, hepatoprotective, antioxidant, and antidiarrheal, of *A. paniculata* are well documented [6,7,8,9,10,11,12,13,14,15,16,17,18,19,20,21]. Therefore, it has been listed as an essential medicinal plant species by the Indian National Medicinal Plants Board [22]. Furthermore, it is also reported that andrographolide inhibits the main protease of novel coronavirus (SARS-CoV-2) [23,24,25]. However, increasing the amount of andrographolide in *A. paniculata* proves to be a major challenge due to its naturally low concentration in the plant.

The NAC transcription factor (TF) is one of the largest plant-specific transcription factors, determining various processes such as growth, development, the synthesis of secondary metabolites, and stress responses. The NAC name has been derived from *Petunia* as NAM, and *Arabidopsis* as ATAF1, ATAF2, and CUC2 [26,27]. The conserved NAC domain contains five sub-domains, which are situated at the N-terminal portion of the NAC protein and help in DNA binding, nuclear localization, and the construction of homodimers and heterodimers with other NAC proteins [28,29,30], while the C-terminal portion of the NAC protein is the TR domain, which is usually not conserved, and may work as an activator or repressor [31]. In response to stress, NAC transcription factors play a crucial role in regulating the expression of various target genes. This regulation occurs by binding NAC TFs to a specific DNA sequence (CATGTG motif), typically located in the promoter region of the target gene [32].

The members of the NAC family are reported to have a key role in transcriptional reprogramming linked with plant species developmental and stress responses such as the development of lateral root, hormonal signaling, seed development, flower morphogenesis, secondary cell wall development, drought, cold stress, and salt stress [33,34,35,36,37,38,39,40,41]. Phytohormones are plant growth regulators which induce the key signals that regulate the series of physiological processes and secondary metabolite production in plants in response to various stresses [42,43]. Abscisic acid (ABA) is a key regulator in plant responses to abiotic stress, orchestrating physiochemical changes. On the other hand, methyl jasmonate (MeJA) plays a pivotal role in activating defense responses against pathogens and pests, stimulating the production of protective compounds [42,44,45,46,47,48]. Earlier reports demonstrated that abiotic stress could induce the expression of NAC transcription factors in other crops like *Oryza sativa* and *Arabidopsis thaliana*. For instance, NAC transcription factors (*AtNAC072*, *AtNAC55*, and *AtNAC019*) are induced by ABA, high temperatures, and drought stress in *A. thaliana* [42]. Previous reports suggest that the NAC transcription factor modulates the expression of genes related to the secondary metabolite pathway, leading to the overproduction of secondary metabolites having medicinal values [49,50,51]. The coordinated regulation of secondary metabolite production via NAC transcription factors is an emerging field for the analysis of the net output, evoking signals, and controlling the signaling steps; examples include artemisinin and dihydroartemisinic acid content production in *Artimisia annua* [49], monoterpene production in *Actinidia arguta* [52], carotenoid production in *Solanum lycopersicum* [53], and caffeine production in *Camellia sinensis* [50]. The overexpression of the NAC transcription factor from *A. annua* (*AaNAC01*) induced the biosynthesis of artemisinin, a sesquiterpene lactone class of secondary metabolite in *A. annua* [49]. A similar report in *Camellia sinensis* also suggests that the NAC transcription factor modulates the expression of caffeine synthase gene (*yhNMT1*), which leads to a higher accumulation of caffeine in the plant [50].

Despite the availability of the reference genome of *A. paniculata* [54], the exploration of NAC transcription factors within *A. paniculata* remains an underexplored domain, with only a few studies to date [55,56]. Being a medicinally important plant, more efforts are needed for the detailed study of the NAC family. The present investigation is being carried out for the identification, full-length isolation, quantitative real-time PCR-based expression analysis, and heterologous expression of *ApNAC83*, *ApNAC21 22*, and *ApNAC02* transcription factors. These transcription factors were characterized in silico for protein structure, subcellular localization, structure-based function, and regulatory sequence. Estimations of diterpenoids (andrographolide, neo-andrographolide, and 14-deoxyandrographolide and andrographolide glycoside) from the leaves of abscisic acid-treated plants at different time intervals and the expression analysis of *ApNAC83*, *ApNAC21 22*, and *ApNAC02* genes during andrographolide biosynthesis were also undertaken. The potential of these ApNAC genes to modulate the expression of terpene biosynthesis genes was also assessed in a heterologous system (*Nicotiana benthamiana*).

## 2. Materials and Methods

### 2.1. Retrieval and Identification of Core Fragments of NAC Genes

Sequences for NAC domain-contained transcripts were retrieved from the transcript and SSR database of *A. paniculata* [57]. Ten transcripts were selected to identify the NAC gene’s core fragment and were confirmed using an expasy translator for amino acid frame identification (https://web.expasy.org/translate/; accessed on 5 May 2023). The identified amino acid frames were subjected to basic local alignment search tool protein (BLASTP) for homology confirmation with the NAC protein (https://blast.ncbi.nlm.nih.gov/Blast.cgi?PAGE=Proteins; accessed on 5 May 2023). The gene-specific primers (GSP) were designed using the NEB Tm calculator (https://tmcalculator.neb.com/#!/main; accessed on 11 June 2023) and the web tool primer blast. The sequence information of gene-specific primers is given in Table S1.

### 2.2. Plant Material

The seeds of *A. paniculata* genotype IC-342134 were collected from the National Gene bank, ICAR-National Bureau of Plant Genetic Resources, New Delhi, India. Subsequently, the seeds were sown in a germination tray, and 15-day-old seedlings were transplanted to pots at the National Phytotron Facility, ICAR-Indian Agricultural Research Institute, New Delhi. For the RACE experiment, the healthy leaves were collected from the young plant and stored in a deep freezer (−80 °C) until further use. Root, stem, and leaf tissues of the plant were also collected and stored in the deep freezer for tissue-specific expression analysis. *N. benthamiana* plants were grown in a growth chamber, and five-week-old plants were used for the transient expression study.

### 2.3. Total RNA Isolation and cDNA Synthesis

The total RNA from the leaf tissue of *A. paniculata* was isolated using the PureLink RNA Mini Kit (Thermo Fisher Scientific, Waltham, MA, USA). After that, DNase treatment was performed using the TURBO DNA-free Kit (Thermo Fisher Scientific, USA). Subsequently, the quantity and quality of isolated RNA were checked using a Nanodrop Spectrophotometer (Thermo Fisher Scientific, USA), followed by gel electrophoresis. The high-quality RNA (1 μg) was used to synthesize cDNA using the SuperScript™ III cDNA synthesis kit (Thermo Fisher Scientific, USA).

### 2.4. PCR Amplification and Cloning of Core Fragments

For the core fragment amplification of *NAC83*, the PCR reaction consisting of 1 μL diluted cDNA (1:10), 1 μL 10× buffer, 1 μL 2.5 mM MgCl_2_, 1 μL 10 mM dNTPs, 0.5 μL of each CoreN1 primer (10 nmol), 0.2 μL *Ex Taq DNA polymerase* (Takara, Kusatsu, Shiga, Japan), and 5.8 μL ultra-pure distilled water produced the final volume of 10 μL. The PCR reaction was performed in a thermal cycler (G-storm, Middlesbrough, UK) with the initial denaturation at 94 °C for 4 min, followed by 35 cycles of denaturation at 94 °C for 30 s; primer annealing at 58 °C for 30 s; DNA synthesis at 72 °C for 1 min; and a final extension at 72 °C for 10 min. The amplicons were separated using 1.5% agarose (Lonza, Allendale, NJ, USA) gel. The gel image was captured using the gel documentation system (Syngene, Cambridge, UK), and the desired single band was excised. Furthermore, the DNA fragment was eluted using a gel extraction kit (Qiagen, Hilden, Germany), ligated into a pCR2.1 TA cloning vector (Thermo Fisher Scientific, Waltham, MA, USA), and incubated at 16 °C overnight. The ligated product was further transformed into DH5α competent cells, and the transformed product was uniformly spread on IPTG/X-Gal LBA (Luria Bertani Agar) plates. The plates were allowed to grow at 37 °C overnight. Colony PCR screened the positive clones, and a total of 5 positive clones were selected for plasmid isolation and then sequenced (Macrogen Inc., Incheon, Republic of Korea) using the Sanger sequencing method. Then, the vector sequence was trimmed out from the total sequence generated, and multiple sequence alignment was completed using the clustal omega tool (https://www.ebi.ac.uk/Tools/msa/clustalo/; accessed on 16 July 2023) in order to find out the overlapping regions in the sequence generated from five sequenced plasmids. The overlapped nucleotide sequence was used to design gene-specific primers (GSP) for RACE and real-time PCR. One outer and one inner antisense GSP primer for 5′ RACE, one sense GSP primer for 3′ RACE, and one primer pair were designed for real-time PCR using the primer3plus program. Similarly, the core fragment of *NAC21 22* and *NAC02* was isolated, cloned, and sequenced, and the primers for RACE and real-time PCR were designed. All the primers used in RACE PCR and expression analyses are given in Table S1.

### 2.5. 5′ and 3′ RACE and Sequencing of Products

The isolated RNA was used to synthesize 5′ RACE- and 3′ RACE-ready cDNA using a 5′ RACE kit (Thermo Fisher Scientific, USA) and a 3′ RACE kit (Thermo Fisher Scientific, USA), respectively, following the manufacturer’s protocol. Furthermore, 5′ RACE-ready cDNA was primarily amplified using the adaptor abridged primer (AAP) and antisense outer GSP (primer ID-5N1GSP1OP) following the PCR program as follows: initial denaturation at 94° for 3 min, followed by 38 cycles of denaturation at 94° for 30 s, annealing at 55° for 30 s, extension at 72° for 1 min, and a final extension at 72° for 10 min (Table S1). The primary PCR product was re-amplified using the abridged universal amplification primer (AUAP) and inner GSP (primer ID-5N1GSP2IP) at different annealing temperatures, ranging from 55.1 °C to 63.4 °C (Table S1). The secondary PCR product was checked on 1.5% agarose gel, and the expected band was excised. Furthermore, 3′ RACE-ready cDNA was amplified in a thermal cycler using 3′ GSP (primer ID-3N1GSP) and the adaptor primer (AP) according to the above-described PCR program, except for Ta (58.1 °C to 68.2 °C for 30 s) (Table S1). The PCR product was separated on 1.5% agarose gel, and the expected product was excised. Furthermore, both 5′ and 3′ RACE PCR products were extracted using a gel extraction kit (Qiagen, Germany). The eluted PCR products were ligated to the TA cloning vector and transformed into *Escherichia coli* (DH5α) competence cells. The positive clones were screened using colony PCR. Plasmids were isolated from positive clones using a plasmid isolation kit (Qiagen, Germany) and subjected to Sanger sequencing (Macrogen Inc., Incheon, Republic of Korea). Similarly, 5′ and 3′ RACE of *ApNAC21 22* and *ApNAC02* were performed. The obtained full-length sequence of the genes was submitted to NCBI with accession numbers MW645354 (*ApNAC83*), MZ458369 (*ApNAC21 22*), and MW645355 (*ApNAC02*).

### 2.6. Sequence Analysis

The open reading frame (ORF) finder, NCBI conserved domain search, and SSR Locator (Simple sequence repeats locator) were used to predict the possible ORFs, conserved domain, and SSRs in the full-length sequence of the genes, respectively. The amino acid sequence, molecular weight, and isoelectric point of the protein were obtained using the expasy web tool. The multiple sequence alignment of the amino acid sequences of isolated NACs and other known NAC domain proteins from *A. thaliana*, i.e., *AtNAM* (AT1G52880), *AtATAF1* (AT1G01720) and *AtCUC1* (AT3G15170) was performed to identify conserved regions and potential functional domains. The phylogenetic tree was constructed using the neighbor-joining method with 1000 bootstrap replicates, comprising homologs of *ApNAC83*, *ApNAC21 22*, and *ApNAC02* from different plant species, and NAC TFs characterized at the protein level from *A. thaliana* and *O. sativa* in MEGA 6 software [58]. The 5′ UTR region of the genes was used to predict cis-acting regulatory elements using the plant care database [59].

### 2.7. Protein Structure and Their Function Prediction

The secondary structure of the proteins was predicted using the Phyre2 modelling tool [60]. After that, the I-Tasser (Iterative Threading Assembly Refinement) server was used for the prediction of the tertiary modelling of the proteins, using the ANAC019 protein from *A. thaliana* (PDB ID: 3SWP) as the template [61,62,63]. The molecular function, biological process, and cellular component predictions were carried out using the COFACTOR server and OmicsBox, Valencia, Spain [64,65].

### 2.8. Assay of Andrographolide Content

To induce the biosynthesis of andrographolide, two elicitor treatments, ABA and MeJA, were performed. For the MeJA treatment, MeJA with a concentration of 50 mM was exogenously sprayed on three-week-old seedlings, leaf samples were collected at different periods (0 h, 24 h, and 48 h), and RNA was isolated for the expression analysis of the genes [54,66,67]. For the ABA treatment, 100 μM ABA was sprayed exogenously on five weeks old plants [68,69]. The plants treated with MeJA and ABA were covered with transparent polybags. The leaf samples at 0 h, 6 h, 12 h, and 24 h after the treatment were collected for expression analysis and andrographolide quantification. For andrographolide quantification, leaf samples were shade dried.

### 2.9. Expression Analysis Using Real-Time Quantitative PCR (qPCR)

The total RNA was isolated from fresh leaf tissues (50 mg) of the plant treated with hormones (MeJA and ABA) and collected at the different time intervals, as mentioned in the hormone treatments section. After that, an equal concentration of RNA (500 ng), isolated from the leaves collected at different time intervals under ABA and MeJA, was used to synthesize cDNA. The expression analysis was performed using the CFX96 Real-time system (Bio-Rad Laboratories, Hercules, CA, USA), as described in previous reports [70,71]. Three replicates of each qPCR reaction were performed, and a non-template negative control (NTC) was also performed. The relative expression level was calculated using the 2^−ΔΔCT^ method and considering the actin gene of *A. paniculata* for normalization [55,72]. During the expression analysis under the MeJA and ABA treatments, all the expressions were compared with the control (untreated). The housekeeping gene actin was used as an endogenous control during expression analysis [55]. Based on the literature search, the andrographolide biosynthesis pathways, such as MVA (mevalonate pathway) and MEP (2-C-methyl-D-erythritol 4-phosphate) pathways, were examined. Enzyme genes associated with these pathways, including *HMGR* (XM_051267731.1), *HDS* (XM_051264732.1), *MVK* (XM_051288144.1), *DXS* (NM_001422027.1), and *DXR* (XM_051280679.1), were also selected for expression analysis under ABA treatment [73,74,75,76]. These genes were previously reported to be strongly induced during the higher accumulation of andrographolide content.

### 2.10. Quantification of Andrographolide

The dried leaves were ground to a coarse powder and weighed. The dry weight of the leaf tissue collected at 0 h, 6 h, 12 h, and 24 h post-treatment was 101 mg, 113 mg, 192 mg, and 160.3 mg, respectively. Furthermore, extraction was performed using the percolation (cold extraction) method. Using this method, dried leaf powder was placed in a percolator, filled with methanol, covered, and kept at room temperature. After 24 h, the methanol solvent was collected from the percolator. This process was repeated three times, and the collected methanol fractions were combined and concentrated under reduced pressure using a rotavapor. The final extracted yield was 0.550 mg, 0.516 mg, 0.970 mg, and 0.730 mg for 0 h, 6 h, 12 h, and 24 h post-treatment, respectively. The samples with a concentration 10 mg/mL were prepared in methanol for the estimation of andrographolide. The standards used were andrographolide, neo andrographolide, 14-deoxyandrographolide, and andrographolide glycoside with concentrations of 1 mg/mL. Furthermore, 4 μL of the standard mix was injected during HPLC (Shimadzu Lab Solutions, Kyoto, Japan), and, at different retention time standards, peaks were recorded (Figure S22). Following this, four different extracted samples were injected with 5 µL volume, and the retention peaks were recorded. Standard peaks were used to identify andrographolide, neo andrographolide, 14-deoxyandrographolide, and andrographolide glycoside in the leaf samples. During the HPLC analysis, the flow rate, concentration of the solvent, and total run time for a single HPLC cycle were 0.3 mL/min, 10 mg/mL, and 50 min, respectively. The analysis was conducted using an RP18 column (4.6 mm × 250 mm, 5 μm), which was operated at 30 °C. Mobile phases A and B were water solutions containing 0.1% formic acid and acetonitrile, respectively. The correlation between the expression of isolated NACs and the biosynthesis of diterpenoids was calculated using Microsoft Excel (MS office 2021) [77].

### 2.11. Transient Expression of Isolated ApNAC Genes in N. benthamiana

To determine whether *ApNAC* genes have the potential to change the expression of genes that control the production of andrographolide, we transiently expressed these *ApNAC* genes in tobacco leaves. The constructs were prepared using the pBI121 binary vector by replacing the GUS reporter gene with *ApNAC* genes. For this, the coding sequence (CDS) of *ApNAC83*, *ApNAC21 22*, and *ApNAC02* genes were PCR amplified, cloned into *XbaI* and *SacI* restriction sites, and the constructs were named *pBI121-ApNAC83*; *pBI121-ApNAC21 22*; and *pBI121-ApNAC02*. After confirmation using Sanger sequencing, all the constructs were transformed into *A. tumefaciens* (strain-GV3101) using the freeze–thaw method [78]. The obtained colonies were confirmed using colony PCR, and positive colonies for each construct were inoculated as primary cultures and incubated for 24 h at 28 °C temperature with continuous shaking at 200 rpm. The primary culture was used to inoculate the secondary culture for overnight growth. When the OD reached 0.4–0.6, the bacterial cells were harvested and dissolved in an infiltration buffer (10 mM MES pH 5.6, 10 mM MgCl_2_, 200 µM acetosyringone) and incubated at 28 °C for 4 h. Furthermore, the suspension was infiltrated into the *N. benthamiana* leaves. The infiltrated plants were kept in the dark for 24 h and shifted into light condition for next 24 h. The *N. benthamiana* plants were also infiltrated with empty vector pBI121 and used as a control. After 48 h, the infiltrated leaves were collected for RNA extraction and cDNA synthesis. The success of infiltration in *N. benthamiana* leaves was checked using quantitative real-time PCR and gene-specific primers for the respective gene (Table S1). Furthermore, the HMGR gene (XM_016590135.1) of the MVA pathways of *Nicotiana tabacum* was selected for quantitative expression analysis in infiltrated leaves.

## 3. Results

### 3.1. 5′ and 3′ RACE and Sequence Analysis

A total of ten putative transcript sequences were initially selected to identify the core NAC fragments from the Andrographis transcripts and SSR database [57], using expasy translation and the protein basic local alignment search tool (BLASTP) for homology. Out of these, eight transcripts demonstrated homology with NAC TF of different plant species, revealed from the National Centre for Biotechnology Information (NCBI) database. Furthermore, only three core fragments were PCR amplified for 5′ and 3′ RACE (NAC83, NAC21 22 and NAC02), which were subsequently cloned and sequenced (Table S1 and Figure S1). The 5′ RACE and 3′ RACE experiments were performed and sequenced, resulting in 5′ RACE products of 481 bp, 560 bp, and 676 bp for NAC83, NAC21 22, and NAC02, respectively, and 3′ RACE products of 354 bp, 451 bp, and 494 bp for NAC83, NAC21 22, and NAC02, respectively (Figure S1). The sequence analysis using the GeneMark tool (http://opal.biology.gatech.edu/GeneMark/; accessed on 12 July 2023) and ORF finder (https://www.ncbi.nlm.nih.gov/orffinder/; accessed on 12 July 2023) indicated that *ApNAC83*, *ApNAC21 22*, and *ApNAC02* contain CDS regions of 687 bp, 771 bp, and 840 bp, respectively (Figures S2–S4). Finally, a full-length cDNA sequence of *ApNAC83*, *ApNAC21 22*, and *ApNAC02* was identified, which comprised 1102 bp (5′ UTR-247; 3′ UTR-168 bp; and CDS-687 bp), 996 bp (5′ UTR-178 bp; 3′ UTR-47 bp; and CDS-771 bp), and 1011 bp (5′ UTR-50 bp; 3′ UTR-121 bp; and CDS-840 bp), respectively (Figures S2–S4).

The designation of *ApNAC83*, *ApNAC21 22*, and *ApNAC02* was based on the BLASTX results, which revealed their highest homology with NAC domain-containing proteins from *Sesamum indicum* (XP_011086848.1), *Salvia splendens* (XP_042068496.1), and *S. splendens* (XP_042049427.1), respectively. The obtained full-length sequence of the genes was submitted to NCBI with accession numbers MW645354 (*ApNAC83*), MZ458369 (*ApNAC21 22*), and MW645355 (*ApNAC02*). The *ApNAC83* encodes a protein of 228 amino acids with a molecular weight of 25.59 kDa and an Iso-electric point (pI) of 9.35 (Table S2). The instability index (II), aliphatic index (AI), and grand average of hydropathicity (GRAVY) of *ApNAC83* are 45.11, 61.14, and −0.778, respectively (Table S2). *ApNAC21 22* encodes a protein of 256 amino acids with a molecular weight of 29.16 kDa and a pI of 8.95. The II, AI, and GRAVY values of *ApNAC21 22* are 50.67, 60.94, and −0.589, respectively (Table S2). The *ApNAC02* gene encodes a protein of 279 amino acids, which have a molecular weight of 31.04 kDa and a pI of 7.01. The II, AI, and GRAVY values of *ApNAC02* were 44.43, 66.56, and −0.498, respectively (Table S2). The observed higher II value (greater than 40) and negative GRAVY value of all genes indicated that they are unstable and hydrophilic proteins.

**Figure 1:**
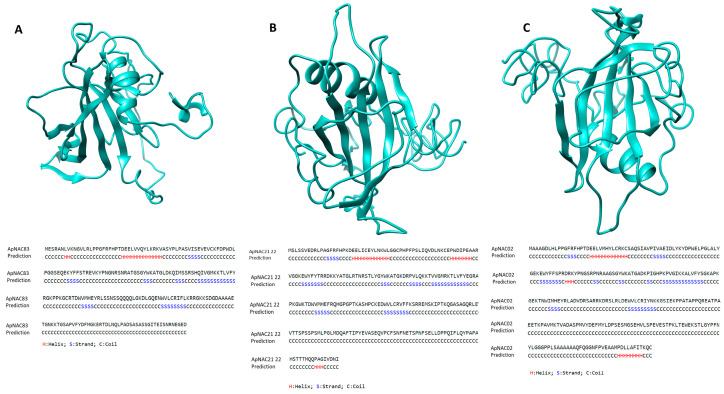
Stereo ribbon cartoon representation of NAC protein structures, generated by i-TASSER and based on homology modelling, and their secondary structure elements corresponding to helices and strands. (**A**) ApNAC83: 18 helix and 39 strands; (**B**) ApNAC21 22: 25 helix and 43 strands; (**C**) ApNAC02: 23 helix and 46 strands. Helix and strands are labelled as H (red) and S (blue), respectively.

The cellular component and subcellular localization prediction indicated that all the genes were mainly found in the nucleus (Figures S5–S10). Several SSRs were predicted in the full-length sequence of the genes. Seven SSRs were detected in the full-length sequence of *ApNAC83*, with four in the 5′ untranslated region (UTR) and three in the coding sequence (CDS). Nine SSRs were detected in *ApNAC21 22*, with five in the 5′ UTR, one in the 3′ UTR, and three in the CDS region. Eight SSRs were detected in *ApNAC02*, with one in the 5′ UTR, one in the 3′ UTR, and six in the CDS. The details of the reported microsatellites are given in Table S3. The upstream region of the genes was predicted to contain various *cis*-acting regulatory elements, particularly ABRE (Abscisic acid responsive element), ABRE3a, ABRE4, ARE, CAAT-box, G-box, STRE (stress responsive element), TATA-box, and W box (*ApNAC83*); AT~TATA box, CAAT-box, G-box, GT1-motif, MSA-like, STRE, TATA-box, and TCCC-motif (*ApNAC21 22*); and GC-motif and DRE2COREZMR (*ApNAC02*). Their position, strand matrix, and putative functions are given in Table S4. The predicted secondary structures of ApNAC83, ApNAC21 22, and ApNAC02 proteins were analyzed. The ApNAC83 protein was found to be composed of 75% coil regions, with α-helix and β-sheet accounting for 7.8% and 17.10%, respectively (Figure 1). Similarly, the ApNAC21 22 protein showed a significant coil region (73.84%), along with α-helix (9.6%) and β-sheet (16.53%) (Figure 1). Likewise, the ApNAC02 protein had a coil region comprising 75.26% of its predicted secondary structure, with α-helix and β-sheet representing 8.24% and 16.48%, respectively (Figure 1).

Overall, all the proteins were mainly composed of coiled regions. *ApNAC83* was predicted to bind with DNA, and amino acid residues *viz*, Gly 97, Thr 96, Val 118, Arg 129, and Lys 166 from the NAC domain *ApNAC83* are active sites for the formation of a channel for DNA binding (Figure S11). The DNA binding pocket of *ApNAC21 22* indicated the presence of amino acid residues, i.e., Gly 99, Thr 98, Lys 129, Thr 130, Phe 161, and Lys 163, while Thr 26, Gly 27, Cys 49, Gly 63, Tyr 98, and Lys 100 for *ApNAC02* are predicted to be active sites for the formation of a major channel for DNA binding (Figure S11).

**Figure 2:**
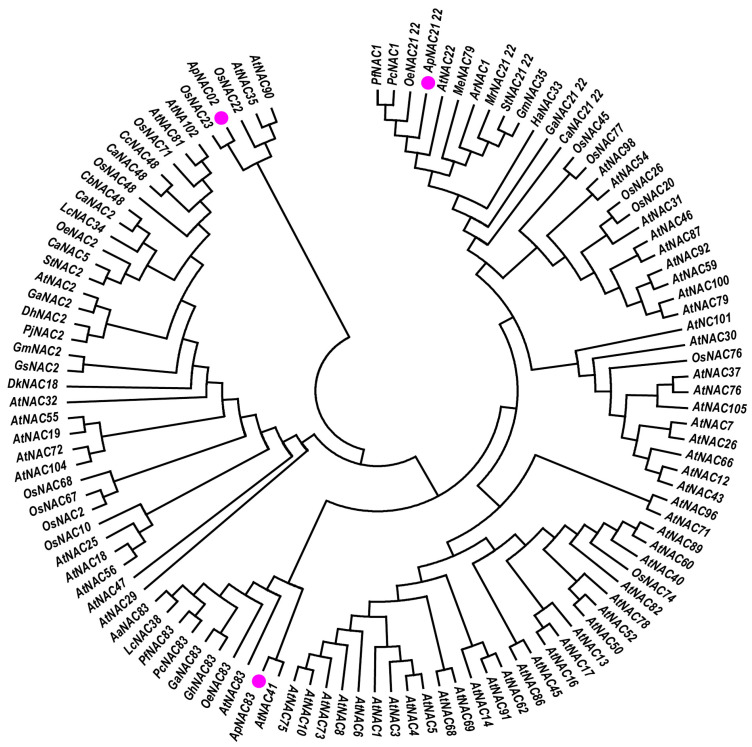
N-J phylogenetic tree of the amino acid sequences of homologs *ApNAC83*, *ApNAC21 22*, and *ApNAC02*. The pink circle indicates the NAC isolated from *A. paniculata*.

The multiple sequence alignment analysis showed the five conserved sub-domains (a–e) and a highly variable domain (C-terminus) in the amino acid sequences of isolated NACs (Figure S12). Phylogenetic analysis indicated that *ApNAC83* is closely related to *AtNAC41* (O22798), *AtNAC83* (Q9FY93), *OeNAC83* (CAA3022015), *GhNAC83* (NP_001313946), *GaNAC83* (KAA3463694), *PcNAC83* (KAH6771146), *PfNAC83* (KAH6809858), *LcNAC38* (UBT01642), and *AaNAC83* (PWA81517). *ApNAC21 22* demonstrated a close relationship with *OeNAC21 22* (CAA2970847), *PcNAC1* (KAH6822852), and *PfNAC1* (KAH6778679). Additionally, *ApNAC02* demonstrated a close relationship to *OsNAC23* (Q6H8A9), *OsNAC22* (Q10S65), *AtNAC35* (Q9ZVP8), and *AtNAC90* (Q9FMR3) (Figure 2).

### 3.2. Structure-Based Function Prediction

The predicted molecular functions of the genes were transcription factor activity and sequence-specific DNA binding as per gene ontology (GO) term (GO:0003700) (Figure S13). The biological process prediction showed that *ApNAC83* and *ApNAC21 22* are involved mainly in the biological process (GO:0006355), transcription process, DNA-templated, cellular process, nitrogen compound metabolic process, cellular metabolic process, and single-organism process, whereas *ApNAC02* is involved in the biological process (GO:050794), i.e., the single-organism process, biological regulation, the regulation of the biological process, and responses to stimuli (Figures S14–S16). Furthermore, the Blast2GO gene ontology classification of NAC gene sequences under the biological process showed that isolated *ApNAC83* and *ApNAC21 22* could be involved in regulating transcription and DNA binding, which is the common function of NAC transcription factors in other plant species as well (Figures S13–S17). Interestingly, *ApNAC02* was predicted to be involved in responses to wounding and the regulation of ABA-activated signaling (Figure S17).

### 3.3. Diterpenoid Profiling

The diterpenoid profiling of leaves collected at different time intervals following ABA treatment was performed using HPLC. The highest andrographolide content (343.12 ng g^−1^ dry weight of leaves) was estimated in leaves 6 h after ABA treatment compared to untreated control (185.67 ng g^−1^ dry weight of leaves). Following this, it was observed that the andrographolide content remained more or less the same at 12 h (326.95 ng g^−1^ dry weight of leaves) and 24 h (332.14 ng g^−1^ dry weight of leaves) (Table 1 and Figures S18–S22). The 14-deoxyandrographolide content was highest in the leaves (41.02 ng g^−1^ dry weight of leaves) at 24 h, and lowest (6.41 ng g^−1^ dry weight of leaves) 12 h after the treatment, while the neoandrographolide content was decreased under ABA treatment, as compared to the untreated control. However, the andrographolide glycoside content remained unchanged under ABA treatment.

### 3.4. Expression Profiling

**Figure 3:**
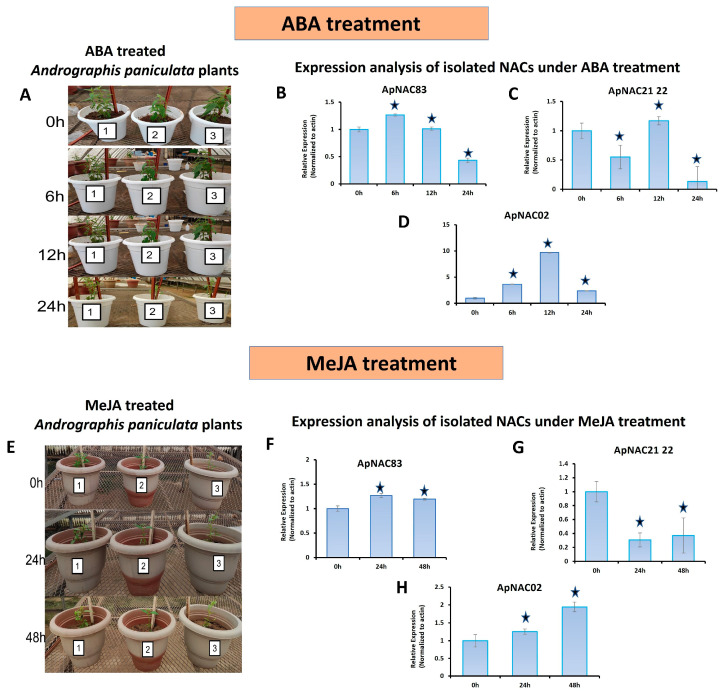
The *A. paniculata* plants used for elicitor treatments and quantitative expression analysis. (**A**) Abscisic acid (ABA)-treated plants; Relative expression of isolated NAC genes in response to ABA treatment at a different time interval (0 h, 6 h, 12 h, and 24 h): (**B**) *ApNAC83*, (**C**) *ApNAC21 22*, (**D**) *ApNAC02*; (**E**) Methyl jasmonate (MeJA)-treated plants; relative expression analysis of isolated NAC genes in response to MeJA treatment at different time intervals (0 h, 24 h, and 48 h): (**F**) *ApNAC83*, (**G**) *ApNAC21 22*, (**H**) *ApNAC02*. Three biological replicates of each treatment were performed. Leaf samples were collected at various time intervals post-treatment: 0 h, 6 h, 12 h, and 24 h after ABA treatment, and 0 h, 24 h, and 48 h after MeJA treatment. Differences were scored as statistically significant at * *p* < 0.05. Asterisk symbols indicate significance.

The untreated tissue-specific expression of the genes showed that *ApNAC21 22* and *ApNAC02* were higher in the leaves than in stems and roots (Figure S23). The expression of *ApNAC83* was higher in the roots than in leaves and stems. The ABA-treated leaf samples showed differential expression for all three isolated genes (Figure 3A–D). The expression of *ApNAC21 22* was downregulated 6 h and 24 h after ABA treatment. The expression of *ApNAC83* was downregulated 24 h after the treatment when compared to control. The expression of *ApNAC02* was upregulated under ABA treatment, reaching a peak at 12 h (9.6-fold), and then declined after 24 h. The expression of *ApNAC21 22* and *ApNAC02* under MeJA treatment was differentially expressed in leaves. The expression of the *ApNAC21 22* was downregulated 24 h and 48 h after MeJA treatment, while the *ApNAC02* expression was upregulated and peaked after 48 h (1.94-fold) (Figure 3E–H). However, no considerable change was observed in the expression of *ApNAC83* under MeJA treatment.

**Figure 4:**
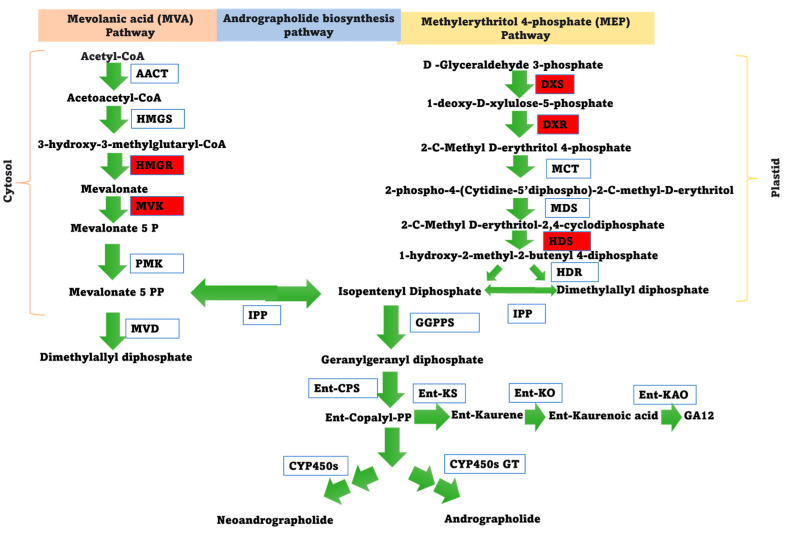
The schematic representation of the proposed andrographolide biosynthesis pathway. AACT is acetyl-CoA acyltransferase; HMGS is 3-hydroxy-3-methylglutaryl-CoA; HMGR is 3-hydroxy-3-methylglutaryl-CoA reductase; MVK is mevalonate kinase; PMK is phosphomevalonate kinase; MVD is mevalonate 5-diphosphate decarboxylase; DXS is 1-deoxy-D-xylulose 5-phosphatesynthase; DXR is 1-deoxy-D-xylulose 5-phosphate reductoisomerase; MCT is MEP cytidylyltransferase; CMK, 2-phospho-4-(cytidine5-diphospho)-2-C-methylerythritol kinase; MDS is 2-C-methy-D-erythritol 2,4-cyclodiphosphate synthase; HDS is hydroxymethylbutenyl 4-diphosphatesynthase; HDR is 4-hydroxy-3-methylbut-2-enyldiphosphatereductase; IPP is isopentenyl diphosphate isomerase; GGPPS is geranylgeranyl diphosphate synthase; ent-CPS is ent-copalyl diphosphate synthase; ent-KS is ent-kaurene synthase; ent-KO is ent-kaurene oxidase; ent-KAO is ent-kaurenoic acid oxidase; CYP450 is cytochrome P450 monooxygenase; GT is glycosyltransferase. The pathway enzyme genes selected during real-time q-PCR are highlighted in red.

The biosynthesis of andrographolide involves the formation of geranylgeranyl diphosphate as a precursor, followed by enzymatic steps leading to the conversion of ent-diterpenol (ent-copalyl-PP) into andrographolide [74]. The schematic representation of the proposed andrographolide pathway is provided in Figure 4. Five genes related to the andrographolide pathway were selected for quantitative expression analysis under ABA and MeJA treatment. The expression of two genes (*DXR* and *HMGR*) were upregulated (>5-fold) under ABA treatment. The *DXR* and *HMGR* genes were upregulated 5.48-fold and 8.6-fold higher after 6 h under ABA treatment, respectively, and then gradually declined (Figure S24). Under the MeJA treatment, the expression of *DXS*, *DXR*, *HDS*, *HMGR*, and *MVK* were upregulated by 2.49-fold, 5.5-fold, 10-fold, 12.3-fold, and 2.2-fold, respectively (Figure S25). The expressions of the *HMGR* gene under both treatments (ABA and MeJA treatment) were observed to be the highest.

### 3.5. Correlation Analysis

A moderate positive correlation (R = 0.54) was observed between the expression levels of *ApNAC02* and the biosynthesis of andrographolide under ABA treatment (Table S5) [79]. In contrast, moderate negative correlations were noted with other metabolites, including neo-andrographolide (R = −0.54), 14-deoxyandrographolide (R = −0.46), and andrographolide glycoside (R = −0.44). A positive correlation (R = 0.34) was observed between the expression levels of *ApNAC21 22* and the biosynthesis of neo-andrographolide, while a negative correlation with andrographolide (R = −0.43) and 14-deoxyandrographolidex (R = −0.91) was observed under ABA treatment [79]. However, no considerable correlation was observed between the expression of *ApNAC21 22* and andrographolide glycoside (R = −0.031). Regarding *ApNAC83*, a positive correlation with the biosynthesis of andrographolide glycoside (R = −0.71) was observed, while a negative correlation was noted with 14-deoxyandrographolide (R = −0.86) under ABA treatment. However, no considerable correlation was observed between *ApNAC83* and andrographolide or neo andrographolide. Notably, andrographolide, the principal secondary metabolite from the plant, exhibited a considerable correlation with *ApNAC02* and *ApNAC21 22*, indicating their involvement in the biosynthesis of andrographolide [79].

### 3.6. Effects of ApNAC Genes on Terpene Biosynthesis Genes in N. benthamiana

**Figure 5:**
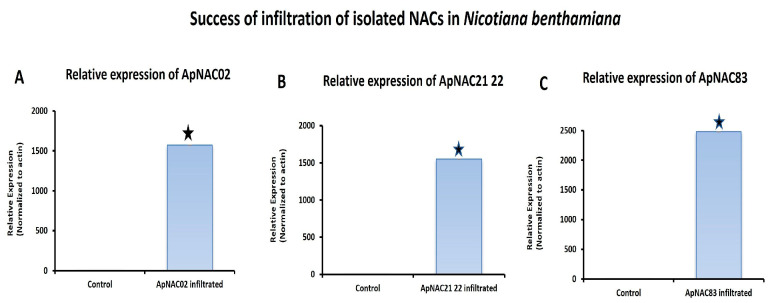
Relative expression of *ApNAC02*, *ApNAC21 22*, and *ApNAC83* in 35S:*ApNAC02*-, 35S:*ApNAC21 22*-, 35S:*ApNAC83*-infiltrated *N. benthamiana* leaves, respectively. All NAC genes were isolated from *A. paniculata* and transiently expressed in *N. benthamiana*. Differences were scored as statistically significant at * *p* < 0.05. Asterisk symbols indicate significance.

**Figure 6:**
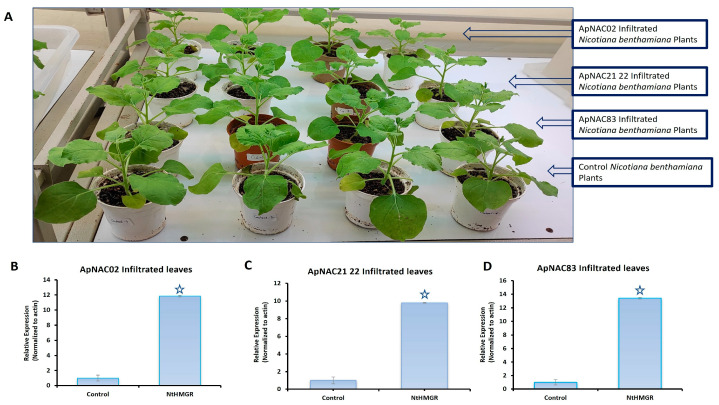
Transient expression of isolated ApNACs in *N. benthamiana* and the relative expression of *NtHMGR* in *35S:ApNACs*-infiltrated leaves. (**A**) *N. benthamiana* plants used for the transient expression of isolated NACs (*ApNAC83*, *ApNAC21 22*, and *ApNAC02*). Control plants were infiltrated with pBI121 empty vectors (35S:GUS). Relative expression of *NtHMGR* in (**B**) *35S:ApNAC02*-, (**C**) *35S:ApNAC21 22*-, (**D**) *35S:ApNAC83*-infiltrated *N. benthamiana* leaves, respectively. All NAC genes were isolated from *A. paniculata* and transiently expressed in *N. benthamiana*. Differences were scored as statistically significant at * *p* < 0.05. Asterisk symbols indicate significance.

The relative expression *ApNAC83*, *ApNAC21 22*, and *ApNAC02* in *35S:ApNAC02*-, *35S:ApNAC21 22*-, and *35S:ApNAC83*-infiltrated tobacco leaves, respectively, served as indicators of successful infiltration in tobacco (Figure 5) to check whether *ApNAC* genes can alter the expression of terpene biosynthesis genes in a heterologous system such as *N. benthamiana*. As per KEGG pathway database, we found that the initial pathway of terpenes biosynthesis is common in *N. benthamiana* and *A. paniculata*. Therefore, we selected andrographolide pathways genes (*DXR* and *HMGR*) which were strongly induced (more than five-fold) under ABA and MeJA treatment in *A. paniculata*. We then predicted *cis*-acting regulatory elements in the promoter region (2 kb) of *DXR* and *HMGR* genes from *A. paniculata* and *N. tabacum* for the presence of the putative NAC-binding site (CATGTG) [32]. The putative NAC-binding site was found in the promoter region of *ApHMGR* and *NtHMGR* (Tables S6 and S7). Therefore, we checked the expression of the *NtHMGR* gene in leaves infiltrated with constructs *35S:ApNAC02*, *35S:ApNAC21 22*, and *35S:ApNAC83*. Interestingly, we found the significant upregulation of the *NtHMGR* gene in *35S:ApNAC02*- (11.8-fold), *35S:ApNAC21 22*- (9.8-fold), and *35S:ApNAC83*-infiltrated (13.4-fold) leaves as compared to 35S:GUS (pBI121)-infiltrated leaves (control) (Figure 6). These results suggest that *ApNAC83*, *ApNAC21 22*, and *ApNAC02* could be potential transcriptional factors which can regulate andrographolide biosynthesis in *A. paniculata*.

## 4. Discussion

Transcription factors are important modulators that could activate the biosynthesis-associated genes at the transcription level to improve the production of bioactive compounds [49,51]. Earlier reports suggest that putative WRKY, AP2/ERF, and bHLH transcription factors from *A. paniculata* were identified using genome or transcriptome data, and their real-time PCR-based expression studies were performed to understand their role in andrographolide biosynthesis [66,80,81]. However, there is a scarcity of research focusing on the molecular and metabolic profiling of the plant [5,54]. This study represents the first instance of isolating the NAC transcription factor from *A. paniculata* and validating its function in *N. benthamiana*.

In the present investigation, the full-length sequences of three members of the NAC transcription factor family (*ApNAC83*, *ApNAC21 22*, and *ApNAC02*) were isolated and their characterization was carried out. The metabolic profiling of the plant was also performed under ABA treatment. The three members of the NAC family were identified, and 3′ UTR and 5′ UTR of these genes were isolated and purified. The upstream region of the genes was used to identify cis-acting regulatory elements. The ABRE (the abscisic acid-responsiveness element), STRE (stress-responsive element), G-box, and W-box regulatory elements were detected in the upstream region of *ApNAC83*. Furthermore, STRE (stress-responsive element) and light-responsive elements like TCCC-motif (TCTCCCT), GT1-motif (GTGTGTGAA), and G-box (CACGAC) were detected in the upstream region of *ApNAC21 22*, whereas GC-motif (CCCCCG) and drought responsive element DRE2COREZMRAB17 (ACCGAC) were detected in *ApNAC02*. The presence of these regulatory elements suggested the regulatory role of isolated NAC genes in the various biological processes, including stress response and secondary metabolite biosynthesis [82]. BLAST2GO indicated that *ApNAC02* could be an ABA-responsive gene. ABA is known to be involved in the plant’s response to abiotic stress via modulating physiochemical changes, while MeJA plays a significant role in the defense response of plants against pathogens and pests by increasing the production of defensive compounds [44,45,46,47,48]. Earlier reports suggest that MeJA and ABA regulate the biosynthesis of secondary metabolites through inducing the expression of genes encoding transcription factors, which then activate the transcription of genes involved in secondary metabolite biosynthesis [49,51,68]. Diterpenoids are a class of natural products (secondary metabolites) found in various plants which have diverse biological activities. NAC transcription factors are known to play important roles in regulating plant development, stress responses, and secondary metabolism, including the biosynthesis of secondary metabolites like diterpenoids. In certain plant species, NAC transcription factors have been found to directly or indirectly regulate the expression of genes involved in diterpenoid biosynthetic pathways. These regulatory mechanisms can control the production of diterpenoids in response to environmental cues, developmental stages, or stress conditions [49,51,68].

Therefore, in the present study, the diterpenoid profiling of leaves and the expression profiling of isolated NAC genes under ABA treatment were performed. Furthermore, the expression analysis of the genes under MeJA treatment and tissue-specific expression analysis were also performed. Earlier, Manzoor et al. [69] elucidated that the exogenous application of ABA accumulates the triterpenoid contents in *Glycyrrhiza glabra* L. The accumulation of triterpenoids was reported within 8 h of exogenous application. Similarly, Anuradha et al. [83] estimated the andrographolide at different daily intervals in *A. paniculata* after the application of ABA foliar spray (5 µM). The andrographolide content was maximally accumulated (110%) ten days after the application. In the present investigation, the ABA foliar spray (100 µM) on the aerial part of the plant was performed, and leaf samples were collected on an hourly basis (0 h, 6 h, 12 h, and 24 h) instead of a daily basis following the treatment. The estimation of diterpenoid contents and expression analysis were performed. The biochemical estimation of diterpenoids using HPLC after an exogenous spray of ABA (100 µM) on the aerial parts of the plant leads to a higher accumulation of andrographolide content (188%) in leaves within 6 h of the treatment, which is much higher than reported by Anuradha et al. (2010) [83]. The present study observed a substantial increase in the concentrations of major compounds andrographolide and 14-deoxyandrographolide by 188% and 360%, respectively, following ABA treatment. Minor compounds, including neo-andrographolide and andrographolide glycoside, were also analyzed in addition to major compounds under ABA treatment. We observed that neoandrographolide was decreased under ABA treatment, while andrographolide glycoside was unchanged. The andrographolide and neo-andrographolide are derived from diterpene lactones and share the same precursor, suggesting a close relationship in their biosynthesis [54]. Andrographolide and 14-deoxyandrographolide are prominent metabolites compared to neo-andrographolide and andrographolide glycoside [54]. In the present study, andrographolide increased under ABA treatment, while neo-andrographolide decreased, indicating competition between the compounds since both are synthesized from the same precursor. The decline in andrographolide glycoside was also observed under ABA treatment. The presence of a major compound like andrographolide can potentially affect the turnover rate of glycoside compounds. Changes in metabolic flux due to environmental factors like ABA/MeJA treatment or salt stress can influence the efficiency of the glycoside derivative turnover [75,84]. Enzymes involved in the formation of andrographolide, neo-andrographolide, 14-deoxyandrographolide, and andrographolide glycoside may regulate the balance between these compounds, thus affecting the turnover rates. Sun et al., 2019 [54] used 50 mM MeJA as an elicitor, observing an increase in andrographolide (266.25%), 14 deoxyandrographolide (173%), and neoandrographolide (480.57%). This suggests that the competition between these compounds can vary depending on various factors, such as the type and concentration of the elicitor, the growth stage of the plant, and the specific plant part used (e.g., whole plant, leaf, stem, or root) [84].

The expression analysis of isolated NAC genes was also performed in ABA-treated plants. Surprisingly, upon hormone induction with ABA, only *ApNAC02* exhibited a strong upregulation (9.6-fold), whereas *ApNAC83* and *ApNAC21 22* were downregulated. Interestingly, a considerable positive correlation (R = 0.54) between the expression of *ApNAC02* and the biosynthesis of andrographolide under ABA treatment was observed [79]. A considerable negative correlation was also observed between the expression level of *ApNAC21 22* and andrographolide [79]. However, no correlation of *ApNAC83* with the andrographolide content under ABA treatment was observed.

Furthermore, the expression analysis of andrographolide pathway-related genes (*HMGR*, *MVK*, *DXS*, *DXR*, and *HDS*) under ABA treatment was also performed [73,74,75,76]. The proteins encoded by HMGR and DXS control the metabolic flux in each pathway (MVA and MEP), influencing enzyme activities, and altering the metabolic pathways’ flow [73,74,75,76]. MVK is an essential enzyme from the cytosolic MVA pathway, which catalyzes the conversion of mevalonate to mevalonate 5 phosphate, which is the precursor of isopentenyl diphosphate. DXR is an important enzyme from the plastid MEP pathway, which catalyzes the conversion of 1-deoxy-D-xylulose-5-phosphate to 2-C-Methyl-D-erythritol 4-phosphate. Furthermore, the HDS enzyme catalyzes the conversion of 2-C-methyl-D-erythritol 2, 4-cyclodiphosphate to 1-hydroxy-2-methyl-2-butenyl 4-diphosphate (HMBPP), which is a key intermediate in terpenoid biosynthesis (Figure 4). HMBPP is further converted to isopentenyl diphosphate with the help of the enzyme HDR. Isopentenyl diphosphate undergoes a series of reactions, then converting to geranylgeranyl diphosphate (GGPP), which is an important precursor utilized in the production of andrographolide/neo-andrographolide. Therefore, DXS, DXR, and HDS from the MEP pathway, as well as HMGR and MVK from the cytosolic MVA pathway, are essential for providing the necessary precursors for andrographolide synthesis. Consequently, these genes were selected for expression analysis under ABA treatment.

The relative expression of *HMGR*, *HDS*, *DXS*, and *DXR* were induced under ABA treatment. The expression of *HMGR*, *DXS*, and *DXR* genes upregulated 6 h after ABA treatment, and then gradually declined. The significant upregulation of these genes and the accumulation of andrographolide content under ABA treatment indicated an association of the genes with the andrographolide content. Earlier studies also reported the upregulation *HMGR*, *DXR*, and *DXS* genes during andrographolide accumulation [73,75,76]. The presence of stress-responsive elements in the promoter region of isolated NACs and the correlation between the expression analysis of the genes and andrographolide profiling under ABA treatment indicate that *ApNAC02* and *ApNAC21 22* genes may be key regulators of andrographolide biosynthesis and stress signaling pathways in *A. paniculata*.

Sun et al. (2019) [54] estimated diterpenoids using HPLC in MeJA-treated Andrographis plants at different time intervals (0 h, 24 h, and 48 h). They found that MeJA treatment induces a higher accumulation of andrographolide content (238%) in 21-day-old seedlings of *A. paniculata* 24 h after the treatment. Wang et al. (2017) [56] performed the real-time qPCR expression analysis of *ApNAC1* under MeJA treatment to decipher the role of the gene in andrographolide biosynthesis. They found that the gene expression was sharply upregulated under MeJA treatment, indicating their role in andrographolide biosynthesis.

Therefore, MeJA treatment was also performed in the present study, and leaf samples were collected at 0 h, 24 h, and 48 h after treatment. However, the estimation of andrographolide was not performed after the treatment, but the expression analysis of isolated genes was undertaken. The expression of two (*ApNAC21 22* and *ApNAC02*) out of three NACs were found to be differentially expressed after MeJA treatment, indicating their role in andrographolide biosynthesis. The expression of *ApNAC21 22* was downregulated, whereas *ApNAC02* (1.9-fold) was upregulated following the MeJA treatment. However, no considerable change in the expression of *ApNAC83* was observed under MeJA treatment. Tissue-specific expression analysis revealed that *ApNAC21 22* and *ApNAC02* expression was higher in leaves when compared with roots. The expression of *ApNAC21 22* and *ApNAC02* consistently showed higher levels in leaves, and previous reports suggested a corresponding higher accumulation of andrographolide content in leaves as well [5]. Higher expression of *ApNAC21 22* and *ApNAC02* in leaves and differential expression under MeJA and ABA treatments suggest that these genes may participate in stress management by inducing andrographolide biosynthesis in leaves. Similarly, previous reports of NAC transcription factors (*NAC02*, *NAC21 22*, and *NAC83*) from another plant species also indicated that *NAC02*, *NAC21 22*, and *NAC83* provide tolerance against abiotic stress in plant [85,86,87,88,89]. As documented by Patil et al. (2014) [85], *AtNAC2* exhibited upregulation during abiotic stress treatment, which is congruent with our findings. Similarly, *NAC02* from the Chinese herb *Salvia miltiorrhiza* demonstrates upregulation in response to MeJA treatment, and is documented to play a role in regulating the biosynthesis of tanshinone content, which is a type of diterpenoid compound [80]. Shinde et al. (2019) [88] noted the upregulation of *PgNAC21* under ABA treatment, but our study found *ApNAC21* to be downregulated, which might be due to species-specific variations.

Earlier reports suggested the upregulation of andrographolide pathway-related genes *HMGR* and *DXR* during andrographolide biosynthesis [73,75,76]. Similarly, terpenes pathway analysis from the KEGG database suggests commonality in the MVA and MEP pathways between *A. paniculata* and *N. tabacum*, as well as their initial terpene pathway enzymes and products. However, the distinct final terpene products indicate species-specific variations in the downstream steps of terpene biosynthesis. In the present study, the expression analysis of *HMGR* and *DXR* was upregulated during andrographolide biosynthesis. Therefore, the promoter region of the genes retrieved from the tobacco genome and the putative NAC-binding site-related cis-acting regulatory element was searched. We found the putative NAC-binding site (CATGTG)-related cis-acting regulatory element in the promoter region of the *HMGR* gene. Additionally, when we examined the relative expression of *NtHMGR* in tobacco leaves infiltrated with *35S:ApNAC02*, we observed an upregulation of the gene when compared to the control. This suggests that *ApNAC02* may induce terpene production in tobacco. However, in addition to the upregulation of the *NtHMGR* gene observed in tobacco leaves infiltrated with *35S:ApNAC02*, we also noticed the upregulation of the gene in tobacco leaves infiltrated with *35S:ApNAC83* and *35S:ApNAC21 22*. It is essential to highlight that these genes did not show strong induction during andrographolide biosynthesis. Therefore, the observed upregulation of *NtHMGR* in tobacco plants infiltrated with *35S:ApNAC83* and 35S:ApNAC21 22 could be attributed to the fact that the target gene originated from a different plant species.

In this study, we observed the following: (i) the functional gene ontology of isolated NACs and the presence of stress-responsive regulatory elements in their upstream regions, (ii) the invariable higher expression of *ApNAC21 22* and *ApNAC02* in the leaf tissue of *A. paniculata*, (iii) the differential expression of *ApNAC02* and *ApNAC21 22* during andrographolide biosynthesis (in response to ABA and MeJA treatment), (iv) the presence of the putative NAC-binding site in the promoter region of the terpene pathway gene (*NtHMGR*), (v) the upregulation of rthe terpene pathway gene (*NtHMGR*) in tobacco plants infiltrated with 35S:ApNAC02 and 35S:ApNAC21 22. Based on these observations, we conclude that NAC transcription factors *ApNAC21 22* and *ApNAC02* may be potential transcriptional factors that can be exploited in order to increase the andrographolide content in *A. paniculata*. However, further studies are necessary to gain a deeper understanding of their involvement in andrographolide biosynthesis.

## Figures and Tables

**Table 1:** The diterpenoids contents in *A. paniculata* leaves after the exogenous application of Abscisic acid (ABA), estimated using the HPLC method.

S.No.	Bioactive Compounds (ng g^−1^)	0 h	6 h	12 h	24 h
1	Andrographolide ^x^	185.67	343.12	326.95	332.14
2	neo andrographolide ^y^	40.31	20.07	23.16	25.02
3	14-deoxyandrographolide ^x^	11.39	14.41	6.41	41.02
4	andrographolide glycoside ^y^	1.2	1.31	1.05	1.06

Where x—major bioactive compound; y—a minor bioactive compound.

## Data Availability

All data supporting the findings of this study are available with the manuscript and supplementary files.

## Supplementary Materials

The following supporting information can be downloaded at https://www.mdpi.com/article/10.3390/genes15040422/s1, Figure S1: Gel image of PCR amplicons: (A) PCR product of core fragment of *ApNAC83*; (B) 5′ RACE PCR amplicon of *ApNAC83*; (C) 3′ RACE PCR amplicon of *ApNAC83*; (D) PCR product of core fragment of *ApNAC21 22*; (E) 5′ RACE PCR amplicon of *ApNAC21 22*; (F) 3′ RACE PCR amplicon of *ApNAC21 22*; (G) PCR product of core fragment of *ApNAC02*; (H) 5′ RACE PCR amplicon of *ApNAC02*; (I) 3′ RACE PCR amplicon of *ApNAC02*. The yellow text (Core fragment, 5′ RACE and 3′ RACE) is used to indicate the ID of the loaded PCR product in the well and M indicates the 100 bp ladder. Where 5′ RACE = 5′ Random amplification of cDNA ends; 3′ RACE = 3′ Random amplification of cDNA ends; Core fragment = PCR product of known partial transcript of the genes.; Figure S2: Full-length cDNA of *ApNAC83*. The full-length cDNA sequence (1102 bp) contains 247 bp 5′ UTR, 168 bp 3′ UTR, and 687 bp CDS region. The Yellow highlighted upstream region is 5′ UTR, Yellow highlighted downstream is 3′ UTR; Figure S3: Full-length cDNA of *ApNAC21 22*. The full-length cDNA sequence (996 bp) contains 178 bp 5′ UTR (Yellow highlighted upstream region), 47 bp 3′ UTR (Yellow highlighted downstream region) and 771 bp CDS region. Start codon and stop codon have been highlighted with green and red color, respectively; Figure S4: Full-length cDNA of *ApNAC02*. The full-length cDNA sequence (1011 bp) contains 50 bp 5′ UTR (Yellow highlighted upstream region), 121 bp 3′ UTR (Yellow highlighted downstream region) and 840 bp CDS region. Start codon and stop codon have been highlighted with green and red color, respectively; Figure S5: Cellular component prediction of *ApNAC83* protein; Figure S6: Subcellular localization of *ApNAC83;* Figure S7: Cellular component prediction of *ApNAC21 22* protein; Figure S8: Subcellular localization of *ApNAC21 22*; Figure S9: Cellular component prediction of *ApNAC02* protein; Figure S10: Sub cellular localization of *ApNAC02;* Figure S11: DNA binding site prediction: A. predicted structure of *ApNAC83* with DNA. B. predicted the structure of *ApNAC21 22* with DNA. C. predicted structure of *ApNAC02* with DNA; Figure S12: Multiple sequence alignment of the deduced amino acid sequences of isolated NACs (*ApNAC02*, *ApNAC21 22*, and *ApNAC83*) along with other known NAC domain proteins from *A. thaliana* (*AtNAM*, *AtATAF1*, and *AtCUC1*). The alignment highlighted the positions of the five highly conserved subdomains (a–e) within the NAC domain, visually represented by lines above the sequences; Figure S13: Molecular function prediction: A. *ApNAC83*. B. *ApNAC21 22*. C. *ApNAC02*; Figure S14: Biological process prediction of *ApNAC83;* Figure S15: Biological process prediction of *ApNAC21 22*; Figure S16: Biological process prediction of *ApNAC02*; Figure S17: Gene ontology classification of NAC gene sequences under biological process; Figure S18: HPLC analysis report of four different bioactive *A. paniculata* under ABA treatment (0 h): where AP-1 is andrographolide, and AP-2 is neo-andrographolide (AP-2), AP-3 is 14-deoxyandrographolide, and AP-4 is andrographolide glycoside. Figure S19: HPLC analysis report of four different bioactive of *A. paniculata* under ABA treatment (6 h): where AP-1 is andrographolide, and AP-2 is neo-andrographolide (AP-2), AP-3 is 14-deoxyandrographolide, and AP-4 is andrographolide glycoside; Figure S20: HPLC analysis report of four different bioactive of *A. paniculata* under ABA treatment (12 h): where AP-1 is andrographolide and AP-2 is neo-andrographolide (AP-2), AP-3 is 14-deoxyandrographolide, and AP-4 is andrographolide glycoside; Figure S21: HPLC analysis report of four different bioactive of *A. paniculata* under ABA treatment (24 h): where AP-1 is andrographolide and AP-2 is neo-andrographolide (AP-2), AP-3 is 14-deoxyandrographolide, and AP-4 is andrographolide glycoside; Figure S22: HPLC analysis report of four different standards used for quantification. Where AP-1 is andrographolide and AP-2 is neo-andrographolide (AP-2), AP-3 is 14-deoxyandrographolide, and AP-4 is andrographolide glycoside; Figure S23: Tissue-specific (root, stem, and leaves) relative expression of isolated genes: (A) *ApNAC83* (B) *ApNAC21 22*; (C) *ApNAC02*. Differences were scored as statistical significance at **p* < 0.05. Asterisk symbols indicate significance; Figure S24: Relative expression of andrographolide pathway related genes in response to ABA treatment at a different time interval (0 h, 6 h, 12 h, and 24 h). Differences were scored as statistical significance at * *p* < 0.05. Asterisk symbols indicate significance; Figure S25: Relative expression of andrographolide pathway related genes in response to MeJA treatment at a different time interval (0 h, 24 h, and 48 h). Differences were scored as statistical significance at * *p* < 0.05. Asterisk symbols indicate significance; Table S1: Details of the primers used in the study; Table S2: Comparative physicochemical parameters of isolated NAC genes; Table S3: List of SSRs, SSR start site, SSR end site, and region where SSR detected in the full length of isolated NAC genes; Table S4: *Cis*-actin regulatory DNA elements, organism, signal sequence, and reported putative functions identified in the promoter region of isolated NAC genes; Table S5: Correlation between the expression of isolated NACs and key metabolites biosynthesis under ABA treatment; Table S6: Cis-acting regulatory elements predicted in promoter region of *ApHMGR*; Table S7: *Cis*-acting regulatory elements predicted in promoter region of *NtHMGR*.

## References

[1] Chandrasekaran C., Thiyagarajan P., Sundarajan K., Goudar K.S., Deepak M., Murali B., Joshua Allan J., Agarwal A. (2009). Evaluation of the genotoxic potential and acute oral toxicity of standardized extract of *Andrographis paniculata* (KalmCold^TM^). Food Chem. Toxicol..

[2] Akbar S. (2011). Andrographis paniculata: A review of pharmacological activities and clinical effects. Altern. Med. Rev. A J. Clin. Ther..

[3] Mishra S.K., Sangwan N.S., Sangwan R.S. (2007). Phcog Rev.: Plant Review *Andrographis paniculata* (Kalmegh): A Review. Rev. Lit. Arts Am..

[4] Nyeem M.A., Mannan M.A., Nuruzzaman M., Kamrujjaman K., Das S. (2017). Indigenous king of bitter (*Andrographis paniculata*): A review. J. Med. Plants Stud..

[5] Garg A., Agrawal L., Misra R.C., Sharma S., Ghosh S. (2015). Andrographis paniculata transcriptome provides molecular insights into tissue-specific accumulation of medicinal diterpenes. BMC Genom..

[6] Abass S., Zahiruddin S., Ali A., Irfan M., Jan B., Haq Q.M.R., Husain S.A., Ahmad S. (2022). Development of Synergy-Based Combination of Methanolic Extract of *Andrographis paniculata* and *Berberis aristata* against *E. coli* and *S. aureus*. Curr. Microbiol..

[7] Kumar R.A., Sridevi K., Kumar N.V., Nanduri S., Rajagopal S. (2004). Anticancer and immunostimulatory compounds from *Andrographis paniculata*. J. Ethnopharmacol..

[8] Bastiana, Widyawaruyanti A., Ilmi H., Tumewu L., Prasetyo B., Hafid A.F., Aryati. (2021). A tablet derived from *Andrographis paniculata* complements dihydroartemisinin-piperaquine treatment of malaria in pregnant mice. J. Basic Clin. Physiol. Pharmacol..

[9] Elasoru S.E., Rhana P., de Oliveira Barreto T., Naves de Souza D.L., Menezes-Filho J.E.R., Souza D.S., Moreira M.V.L., Campos M.T.G., Adedosu O.T., Roman-Campos D. (2021). Andrographolide protects against isoproterenol-induced myocardial infarction in rats through inhibition of L-type Ca^2+^ and increase of cardiac transient outward K^+^ currents. Eur. J. Pharmacol..

[10] Gupta S., Choudhry M.A., Yadava J.N.S., Srivastava V., Tandon J.S. (1990). Antidiarrhoeal activity of diterpenes of *Andrographis paniculata* (kal-megh) against escherichia coli enterotoxin in in vivo models. Pharm. Biol..

[11] Kaur R., Sharma P., Gupta G.K., Ntie-Kang F., Kumar D. (2020). Structure-activity-relationship and mechanistic insights for anti-HIV natural products. Molecules.

[12] Lee D., Baek C.Y., Hwang J.H., Kim M.Y. (2020). *Andrographis paniculata* Extract Relieves Pain and Inflammation in Monosodium Iodoacetate-Induced Osteoarthritis and Acetic Acid-Induced Writhing in Animal Models. Process.

[13] Loh S., Tsai Y., Huang S., Yu T., Kuo P., Chao S.C., Chou M.F., Tsai C.S., Lee S.P. (2020). Effects of andrographolide on intracellular ph regulation, cellular migration, and apoptosis in human cervical cancer cells. Cancers.

[14] Mussard E., Cesaro A., Lespessailles E., Legrain B., Berteina-Raboin S., Toumi H. (2019). Andrographolide, A Natural Antioxidant: An Update. Antioxidants.

[15] Ogundola A.F., Akhigbe R.E., Saka W.A., Adeniyi A.O., Adeshina O.S., Babalola D.O., Akhigbe T.M. (2021). Contraceptive potential of *Andrographis paniculata* is via androgen suppression and not induction of oxidative stress in male Wistar rats. Tissue Cell.

[16] Rajanna M.P., Bharathi B.K., Shivakumar B.R., Deepak M., Prashanth D., Prabakaran D., Vijayabhaskar T., Arun B. (2021). Immunomodulatory effects of *Andrographis paniculata* extract in healthy adults—An open-label study. J. Ayurveda Integr. Med..

[17] Sharma A., Singh R.T., Sehgal V., Handa S.S. (1991). Antihepatotoxic activity of some plants used in herbal formulations. Fitoterapia.

[18] Sharma V., Qayum A., Kapoor K.K., Mukherjee D., Singh S.K., Dhar M.K., Kaul S. (2022). Synthesis of 14-deoxy-benzylidene-8,17-epoxy-diene-andrographolide derivatives and evaluation of their anticancer activities. J. Indian Chem. Soc..

[19] Tohkayomatee R., Reabroi S., Tungmunnithum D., Parichatikanond W., Pinthong D. (2022). Andrographolide Exhibits Anticancer Activity against Breast Cancer Cells (MCF-7 and MDA-MB-231 Cells) through Suppressing Cell Proliferation and Inducing Cell apoptosis via inactivation of ER-α receptor and PI3K/AKT/mTOR signaling. Molecules.

[20] Trivedi N.P., Rawal U.M. (2001). Hepatoprotective and antioxidant property of Andrographis paniculata (Nees) in BHC induced liver damage in mice. Indian J. Exp. Biol..

[21] Visen P.K., Saraswat B., Vuksan V., Dhawan B.N. (2007). Effect of andrographolide on monkey hepatocytes against galactosamine induced cell toxicity: An in-vitro study. J. Complement. Integr. Med..

[22] Verma H., Negi M.S., Mahapatra B.S., Shukla A., Paul J. (2019). Evaluation of an emerging medicinal crop Kalmegh [*Andrographis paniculata* (Burm. F.) Wall. Ex. Nees] for commercial cultivation and pharmaceutical & industrial uses: A review. Pharmacogn. Phytochem..

[23] Kanjanasirirat P., Suksatu A., Manopwisedjaroen S., Munyoo B., Tuchinda P., Jearawuttanakul K., Seemakhan S., Charoensutthivarakul S., Wongtrakoongate P., Rangkasenee N. (2020). High-content screening of Thai medicinal plants reveals Boesenbergia rotunda extract and its component Panduratin A as anti-SARS-CoV-2 agents. Sci. Rep..

[24] Sa-Ngiamsuntorn K., Suksatu A., Pewkliang Y., Thongsri P., Kanjanasirirat P., Manopwisedjaroen S., Charoensutthivarakul S., Wongtrakoongate P., Pitiporn S., Chaopreecha J. (2021). Anti-SARS-CoV-2 activity of *Andrographis paniculata* extract and its major component Andrographolide in human lung epithelial cells and cytotoxicity evaluation in major organ cell representatives. Nat. Prod..

[25] Shi T.H., Huang Y.L., Chen C.C., Pi W.C., Hsu Y.L., Lo L.C., Chen W.Y., Fu S.L., Lin C.H. (2020). Andrographolide and its fluorescent derivative inhibit the main proteases of 2019-nCoV and SARS-CoV through covalent linkage. Biochem. Biophys. Res. Commun..

[26] Aida M., Ishida T., Fukaki H., Fujisawa H., Tasaka M. (1997). Genes involved in organ separation in Arabidopsis: An analysis of the cup-shaped cotyledon mutant. Plant Cell.

[27] Nakashima K., Takasaki H., Mizoi J., Shinozaki K., Yamaguchi-Shinozaki K. (2012). NAC transcription factors in plant abiotic stress responses. Biochim. Biophys. Acta-Gene Regul. Mech..

[28] Duval M., Hsieh T.F., Kim S.Y., Thomas T.L. (2002). Molecular characterization of AtNAM: A member of the Arabidopsis NAC domain super family. Plant Mol. Biol..

[29] Ernst H.A., Nina Olsen A., Skriver K., Larsen S., Lo Leggio L. (2004). Structure of the conserved domain of ANAC, a member of the NAC family of transcription factors. EMBO Rep..

[30] Olsen A.N., Ernst H.A., Leggio L.L., Skriver K. (2005). DNA-binding specificity and molecular functions of NAC transcription factors. Plant Sci..

[31] Liu X., Wang T., Bartholomew E., Black K., Dong M., Zhang Y., Ren H. (2018). Comprehensive analysis of NAC transcription factors and their expression during fruit spine development in cucumber (*Cucumis sativus* L.). Horti. Res..

[32] Nuruzzaman M., Sharoni A.M., Kikuchi S. (2013). Roles of NAC transcription factors in the regulation of biotic and abiotic stress responses in plants. Front. Microbiol..

[33] Guo S., Dai S., Singh P.K., Wang H., Wang Y., Tan J.L., Wee W., Ito T.A. (2018). A membrane-bound NAC-like transcription factor OsNTL5 represses the flowering in *Oryza sativa*. Front. Plant Sci..

[34] Hao Y.J., Wei W., Song Q.X., Chen H.W., Zhang Y.Q., Wang F., Zou H.F., Lei G., Tian A.G., Zhang W.K. (2011). Soybean NAC transcription factors promote abiotic stress tolerance and lateral root formation in transgenic plants. Plant J..

[35] He X.J., Mu R.L., Cao W.H., Zhang Z.G., Zhang J.S., Chen S.Y. (2005). AtNAC2, a transcription factor downstream of ethylene and auxin signaling pathways, is involved in salt stress response and lateral root development. Plant J..

[36] Hou X.M., Zhang H.F., Liu S.Y., Wang X.K., Zhang Y.M., Meng Y.C., Luo D., Chen R.G. (2020). The NAC transcription factor CaNAC064 is a regulator of cold stress tolerance in peppers. Plant Sci..

[37] Jian W., Zheng Y., Yu T., Cao H., Chen Y., Cui Q., Xu C., Li Z. (2021). SlNAC6, A NAC transcription factor, is involved in drought stress response and reproductive process in tomato. J. Plant Physiol..

[38] Sablowski R.W., Meyerowitz E.M. (1998). A homolog of NO APICAL MERISTEM is an immediate target of the floral homeotic genes APETALA3PISTILLATA. Cell.

[39] Wang J., Wang Y., Zhang J., Ren Y., Li M., Tian S., Yu Y., Zuo Y., Gong G., Zhang H. (2021). The NAC transcription factor ClNAC68 positively regulates sugar content and seed development in watermelon by repressing ClINV and ClGH3.6. Hortic. Res..

[40] Wang S., Huang J., Wang X., Dang H., Jiang T., Han Y. (2019). Expression analysis of the NAC transcription factor family of populus in response to salt stress. Forests.

[41] Zhong R., Demura T., Ye Z.H. (2006). SND1, a NAC domain transcription factor, is a key regulator of secondary wall synthesis in fibers of Arabidopsis. Plant Cell.

[42] Tran L.S.P., Nakashima K., Sakuma Y., Simpson S.D., Fujita Y., Maruyama K., Fujita M., Seki M., Shinozaki K., Yamaguchi-Shinozaki K. (2004). Isolation and functional analysis of arabidopsis stress-inducible NAC transcription factors that bind to a drought-responsive cis-element in the early responsive to dehydration stress 1 promoter. Plant Cell.

[43] Fujita M., Fujita Y., Noutoshi Y., Takahashi F., Narusaka Y., Yamaguchi-Shinozaki K., Shinozaki K. (2006). Crosstalk between abiotic and biotic stress responses: A current view from the points of convergence in the stress signaling networks. Curr. Opin. Plant Biol..

[44] Okada K., Abe H., Arimura G.I. (2015). Jasmonates Induce Both Defense Responses and Communication in Monocotyledonous and Dicotyledonous Plants. Plant Cell Physiol..

[45] Pieterse C.M., Van Der Does D., Zamioudis C., Leon-Reyes A., Van Wees S.C. (2012). Hormonal modulation of plant immunity. Annu. Rev. Cell Dev. Biol..

[46] Popko J., Hänsch R., Mendel R.R., Polle A., Teichmann T. (2010). The role of abscisic acid and auxin in the response of poplar to abiotic stress. Plant Biol..

[47] Sirichandra C., Davanture M., Turk B.E., Zivy M., Valot B., Leung J., Merlot S. (2010). The arabidopsis ABA-activated kinase OST1 phosphorylates the bZIP transcription factor ABF3 and creates a 14-3-3 binding site involved in its turnover. PLoS ONE.

[48] Wilkinson S., Davies W.J. (2010). Drought, ozone, ABA and ethylene: New insights from cell to plant to community. Plant Cell Environ..

[49] Lv Z., Wang S., Zhang F., Chen L., Hao X., Pan Q., Fu X., Li L., Sun X., Tang K. (2016). Overexpression of a Novel NAC Domain-Containing Transcription Factor Gene (AaNAC1) Enhances the Content of Artemisinin and Increases Tolerance to Drought and Botrytis cinerea in Artemisia annua. Plant Cell Physiol..

[50] Ma W., Kang X., Liu P., She K., Zhang Y., Lin X., Li B., Chen Z. (2022). The NAC-like transcription factor CsNAC7 positively regulates the caffeine biosynthesis-related gene yhNMT1 in *Camellia sinensis*. Hortic. Res..

[51] Kumar R., Das S., Mishra M., Choudhury D.R., Sharma K., Kumari A., Singh R. (2021). Emerging roles of NAC transcription factor in medicinal plants: Progress and prospects. 3 Biotech.

[52] Nieuwenhuizen N.J., Chen X., Wang M.Y., Matich A.J., Perez R.L., Allan A.C., Green S.A., Atkinson R.G. (2015). Natural variation in monoterpene synthesis in kiwifruit: Transcriptional regulation of terpene synthases by NAC and ETHYLENE-INSENSITIVE3-like transcription factors. Plant Physiol..

[53] Zhu M., Chen G., Zhou S., Tu Y., Wang Y., Dong T., Hu Z. (2014). A new tomato NAC (NAM ATAF1/2/CUC2) transcription factor, SlNAC4, functions as a positive regulator of fruit ripening and carotenoid accumulation. Plant Cell Physiol..

[54] Sun W., Leng L., Yin Q., Xu M.M., Huang M., Xu Z., Zhang Y., Yao H., Wang C., Xiong C. (2019). The genome of the medicinal plant *Andrographis paniculata* provides insight into the biosynthesis of the bioactive diterpenoid neoandrographolide. Plant J..

[55] Kumar R., Kumar C., Jain R., Maurya A., Kumar A., Kumari A., Singh R. (2022). Molecular cloning and In-silico characterization of NAC86 of Kalmegh (*Andrographis paniculata*). Indian J. Hortic..

[56] Wang J., Qi M.D., Guo J., Shen Y., Lin H.X., Huang L.Q. (2017). Cloning, subcellular localization, and heterologous expression of ApNAC1 gene from Andrographis paniculata. Zhongguo Zhongyao Zazhi China J. Chin. Mat. Med..

[57] Singh R., Singh A., Mahato A.K., Paliwal R., Tiwari G., Kumar A. (2023). De Novo Transcriptome Profiling for the Generation and Validation of Microsatellite Markers, Transcription Factors, and Database Development for *Andrographis paniculata*. Int. J. Mol. Sci..

[58] Tamura K., Stecher G., Peterson D., Filipski A., Kumar S. (2013). MEGA6: Molecular evolutionary genetics analysis version 6.0. Mol. Biol. Evol..

[59] Lescot M., Déhais P., Thijs G., Marchal K., Moreau Y., Van De Peer Y., Rouzé P., Rombauts S. (2002). PlantCARE, a database of plant cis-acting regulatory elements and a portal to tools for in silico analysis of promoter sequences. Nucleic Acids Res..

[60] Kelley L.A., Mezulis S., Yates C.M., Wass M.N., Sternberg M.J. (2015). The Phyre2 web portal for protein modeling, prediction and analysis. Nat. Protoc..

[61] Roy A., Kucukural A., Zhang Y. (2010). I-TASSER: A unified platform for automated protein structure and function prediction. Nat. Protoc..

[62] Yang J., Yan R., Roy A., Xu D., Poisson J., Zhang Y. (2014). The I-TASSER suite: Protein structure and function prediction. Nat. Methods.

[63] Yang J., Zhang Y. (2015). I-TASSER server: New development for protein structure and function predictions. Nucleic Acids Res..

[64] Roy A., Yang J., Zhang Y. (2012). COFACTOR: An accurate comparative algorithm for structure-based protein function annotation. Nucleic Acids Res..

[65] Zhang C., Freddolino P.L., Zhang Y. (2017). COFACTOR: Improved protein function prediction by combining structure, sequence and protein-protein interaction information. Nucleic Acids Res..

[66] Yao W., An T., Xu Z., Zhang L., Gao H., Sun W., Liao B., Jiang C., Liu Z., Duan L. (2020). Genomic-wide identification and expression analysis of AP2/ERF transcription factors related to andrographolide biosynthesis in *Andrographis paniculata*. Ind. Crops Prod..

[67] Zhang R., Chen Z., Zhang L., Yao W., Xu Z., Liao B., Duan L., Ji A. (2021). Genomic Characterization of WRKY Transcription Factors Related to Andrographolide Biosynthesis in *Andrographis paniculata*. Front. Genet..

[68] Zhong Y., Li L., Hao X., Fu X., Ma Y., Xie L., Shen Q., Kayani S., Pan Q., Sun X. (2018). AaABF3, an abscisic acid–Responsive transcription factor, positively regulates artemisinin biosynthesis in artemisia annua. Front. Plant Sci..

[69] Manzoor M.M., Goyal P., Pandotra P., Dar M.S., Dar M.J., Misra P., Gupta A.P., Vishwakarma R.A., Ahuja A., Dhar M.K. (2021). Transcriptome-wide identification of squalene epoxidase genes from *Glycyrrhiza glabra* L.: Expression analysis and heterologous expression of GgSQE1 suggest important role in terpenoid biosynthesis. Protoplasma.

[70] Awasthi P., Gupta A.P., Bedi Y.S., Vishwakarma R.A., Gandhi S.G. (2016). Mannitol stress directs flavonoid metabolism toward synthesis of flavones via differential regulation of two cytochrome P450 monooxygenases in coleus forskohlii. Front. Plant Sci..

[71] Manzoor M.M., Goyal P., Gupta A.P., Khan S., Jaswal P., Misra P., Pandotra P., Ahuja A., Vishwakarma R.A., Gupta S. (2020). Chemical and real-time based analysis revealed active gene machinery of glycyrrhizin biosynthesis and its accumulation in the aerial tissues of in-vitro regenerated *Glycyrrhiza glabra* L. Plant Growth Regul..

[72] Livak K.J., Schmittgen T.D. (2001). Analysis of relative gene expression data using real-time quantitative PCR and the 2-ΔΔCT method. Methods.

[73] Jha Z., Sharam S.N., Sharma D.K. (2011). Differential expression of 3-hydroxy-3-methylglutaryl-coenzyme A reductase of *Andrographis paniculata* in andrographolide accumulation. J. Chem. Pharm. Res..

[74] Patel A.A., Shukla Y.M., Kumar S., Sakure A.A., Parekh M.J., Zala H.N. (2020). Transcriptome analysis for molecular landscaping of genes controlling diterpene andrographolide biosynthesis in *Andrographis paniculata* (Burm. f.) Nees. 3 Biotech.

[75] Sharma S.N., Jha Z., Sinha R.K., Geda A.K. (2015). Jasmonate-induced biosynthesis of andrographolide in *Andrographis paniculata*. Physiol. Plant.

[76] Srinath M., Shailaja A., Bindu B.B.V., Giri C.C. (2022). Comparative analysis of biomass, ethrel elicitation, light induced differential MVA/MEP pathway gene expression and andrographolide production in adventitious root cultures of *Andrographis paniculata* (Burm. F.) Nees. Plant Cell Tissue Organ Cult..

[77] Millar N. (2001). Millar Biology statistics made simple using Excel Biology statistics made simple using Excel Spreadsheet programs such as Microsoft Excel can transform the use of statistics in A-level science. Sch. Sci. Rev..

[78] Weigel D., Glazebrook J. (2006). Transformation of agrobacterium using the freeze-thaw method. CSH Protoc..

[79] Ratner B. (2009). The correlation coefficient: Its values range between 1/1, or do they. J. Targeting, Meas. Anal. Mark.

[80] Zhang H., Xu J., Chen H., Jin W., Liang Z. (2021). Characterization of NAC family genes in *Salvia miltiorrhiza* and NAC2 potentially involved in the biosynthesis of tanshinones. Phytochemistry.

[81] Xu J., Xu H., Zhao H., Liu H., Xu L., Liang Z. (2022). Genome-wide investigation of bHLH genes and expression analysis under salt and hormonal treatments in *Andrographis paniculata*. Ind. Crops Prod..

[82] Yamaguchi-Shinozaki K., Shinozaki K. (2006). Transcriptional regulatory networks in cellular responses and tolerance to dehydration and cold stresses. Annu. Rev. Plant Biol..

[83] Anuradha V.E., Jaleel C.A., Salem M.A., Gomathinayagam M., Panneerselvam R. (2010). Plant growth regulators induced changes in antioxidant potential and andrographolide content in *Andrographis paniculata* Wall. ex Nees. Pestic. Biochem. Physiol..

[84] Murthy H.N., Dalawai D. (2021). Biotechnological production of diterpenoid lactones from cell and organ cultures of *Andrographis paniculata*. Appl. Microbiol. Biotechnol..

[85] Patil M., Ramu S.V., Jathish P., Sreevathsa R., Reddy P.C., Prasad T.G., Udayakumar M. (2014). Overexpression of AtNAC2 (ANAC092) in groundnut (*Arachis hypogaea* L.) improves abiotic stress tolerance. Plant Biotechnol. Rep..

[86] Rathnayake K., Garcia T.M., Cushman J.C., Wone B.W. (2019). A Novel NAC83 Transcription Factor from Kalanchoe fedtschenkoi enhances Drought and Salt Tolerance in Arabidopsis. Proceedings of the Plant and Animal Genome XXVII 789 Conference.

[87] Shen J., Lv B., Luo L., He J., Mao C., Xi D., Ming F. (2017). The NAC-type transcription factor OsNAC2 regulates ABA-dependent genes and abiotic stress tolerance in rice. Sci. Rep..

[88] Shinde H., Dudhate A., Tsugama D., Gupta S.K., Liu S., Takano T. (2019). Pearl millet stress-responsive NAC transcription factor PgNAC21 enhances salinity stress tolerance in Arabidopsis. Plant Physiol. Biochem..

[89] Zhao X., Wu T., Guo S., Hu J., Zhan Y. (2022). Ectopic Expression of AeNAC83, a NAC Transcription Factor from *Abelmoschus esculentus*, Inhibits Growth and Confers Tolerance to Salt Stress in Arabidopsis. Int. J. Mol. Sci..

