# Supplementary material for: Isolation, Characterization, and Expression Analysis of NAC Transcription Factor from Andrographis paniculata (Burm. f.) Nees and Their Role in Andrographolide Production
Source: Genes (Basel). 2024 Mar 28;15(4):422. doi: 10.3390/genes15040422 (PMC11049156; doi:10.3390/genes15040422)
Supplement: Supplementary file 1 [file genes-15-00422-s001.zip › Supplementary figures.pdf]

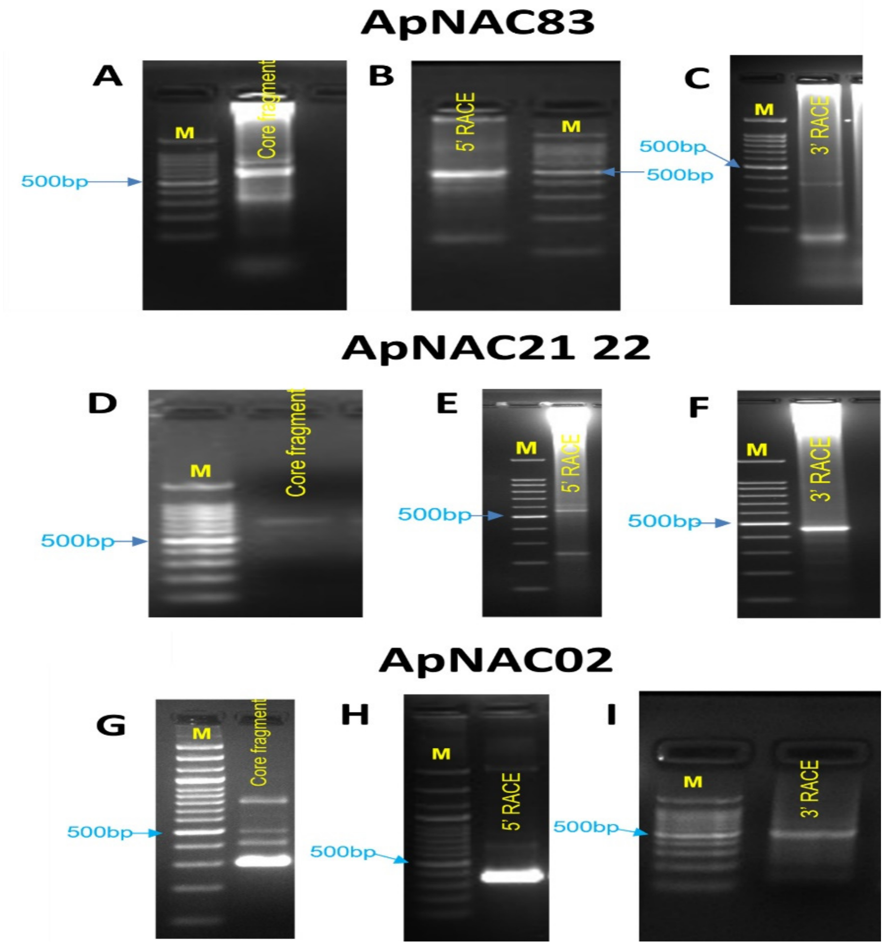

Supplementary Figure S1: Gel image of PCR amplicons: (A) PCR product of core fragment of *ApNAC83*; (B) 5′ RACE PCR amplicon of *ApNAC83*; (C) 3′ RACE PCR amplicon of *ApNAC83*; (D) PCR product of core fragment of *ApNAC21 22*; (E) 5′ RACE PCR amplicon of *ApNAC21 22*; (F) 3′ RACE PCR amplicon of *ApNAC21 22*; (G) PCR product of core fragment of *ApNAC02*; (H) 5′ RACE PCR amplicon of *ApNAC02*; (I) 3′ RACE PCR amplicon of *ApNAC02*. The yellow text (Core fragment, 5′ RACE and 3′ RACE) is used to indicate the ID of the loaded PCR product in the well and M indicates the 100 bp ladder. Where 5′ RACE= 5′ Random amplification of cDNA ends; 3′ RACE=3′ Random amplification of cDNA ends; Core fragment= PCR product of known partial transcript of the genes.

ACCTATCCCCCTCCTCTCTCTCTCTCTCTCTCTCTCTCTCTCTCTCTCTGCTTCTCTTGTTTTATCACGCTGCCCT
GTTCAACTAAGAATAATAGTAATAATAAAAAGGGAGTGTGGGTACGTGTGGGAGGGATTCTTGACCATT
GATGATGAGTTTGAGCTTGTTCTAAGAAAGGGGTTTTTGAGAGGGGGGCTGGAAAAGGCAATCTTGGT
TTCAGTTCCTCTTGTGTCAAATAATATAGAGAATAGATGGAGTCGAGAGCCAACCTTGTCAAGAATGGG
GTGCTGAGACTGCCGCCTGGGTTCAGATTCCACCCAACTGATGAAGAGCTCGTGGTACAATACTTGAAG
CGCAAGGTGGCGTCATATCCTCTGCCGGCTTCTGTAATCTCTGAAGTTGAGGTCTGCAAGTTTGATCCCT
GGGATTTGCCAGGTGGTTCAGAGCAGGAGAAGTATTTTTTCAGCACTAGGGAGGTGAAGTACCCAAAT
GGGAACCGGTCGAATAGGGCCACCGGTTCAGGCTATTGGAAGGCAACAGGACTGGACAAGCAGATAA
TGAGCTCCAGGAGCCACCAGATTGTGGGGATGAAGAAGACTCTAGTGTTCTACAGAGGGAAGCCTCCA
AAGGGTTGCAGAACTGATTGGGTTATGCACGAGTATCGCCTCAGTAGCAATAGTTCCCAACAGCAGCAG
CTGGGCAAGGATTTAGGCCAGGAAAATTGGGTCCTCTGCAGGATTTTCTTGAAGAGGAGAGGCAAGAA
AAGTGATGGTGATGCTGCTGCTGCTGAAACAGGGAACAAGAAGACCGGTTCAGCACCGGTTTTCTATGA
TTTTATGGGTAAGGAGAGGACTGACCTGAATCAACTCCCTGCTGATTCAGCCTCTGCTTCAAGTGGGATC
ACAGAGATTTCAAACAGGAATGAAGGAGAGGACTGACTTGAATCAACTCCCTGCTGATTCAGCCTCTGC
TTCAAGTGGGATCACAGAGATTTCAAACAGGAATGAATCAGAAGAGCTTGAAGAAAGGAGCAGTTGCA
ATAGTCTGCCCACTTCTCTAGCAAGAAAGCAATGCCCCTAGAAAAGTTAAAAAAAAAAAAAAAAAA

Supplementary Figure S2: Full length cDNA of *ApNAC83*. The full-length cDNA sequence (1102bp) contains 247 bp 5′ UTR (Yellow highlighted upstream region), 168 bp 3′UTR (Yellow highlighted downstream) and 687 bp CDS region. Start and stop codon have been highlighted with green and red colour, respectively.

ACGTATGACCTTTCTTCAAACGGTATTATACATCACAGAAATTCCCAGTAGCCCCTCTGTATTGCCCTC

TCCCTTCTCTCCCTCATCTTCCATCCATTCATACATCAATTCACACACACACACACACACGACTATATAT

ATATATGTGTGTGTATATATATATAGCAGGAGCAGAAAGGGATGAGCTTGAGCAGCGTGGAGGATCG

GCTGCCGGCGGGATTCAGGTTCCACCCCAAAGATGAGGAGCTGATATGTGAGTACCTTAACAAATG

GCTAGGTGGTTGTCCTCACCCCTTCCCTTCCTTGATCCAAGTCGACCTCAATAAGTGCGAGCCTTGGG

ACATTCCTGAAGCTGCACGTGTTGGAGGCAAGGAATGGTATTTCTACACCCGGAGAGACAAAAAAT

ACGCGACCGGTCTGAGAACGAACCGGTCGACGCTATACGGGTACTGGAAGGCAACCGGGAAGGAC

CGGCCGGTCCTTCAGAAGAAAACGGTTGTCGGAATGCGAAAGACACTGGTGTTCTATGAGGGCCGG

GCTCCCAAAGGATGGAAAACCGATTGGGTTATGCACGAGTTTCGCCAGCATGGGCCCGGCCCGACG

AAAGCATCACATCCTTGTAAGGAGGACTGGGTGCTATGCAGGGTATTCTTCAAAAGCAGGAGAGAA

ATGAGCAAAATTCCAACCAAACAAGGCGCTAGTGCAGGGCAAAGGCTAGAGGTGACGACATCCCCA

AGTTCTCCATCGATGCTCCCGGGACTAATGGACCAAGCGTTCACGATCCCATACGAAGTCGCCTCTG

AGCAAGTGCCCTGCTTCTCCAATTTCAACGAAACAAGCCCTAACTTCTCTGAGCTCCTGGACCCGCCC

CAAATCTTCTTACAGTACCCAGCTCCTGCTCATTCCACAACGACGATGCAACAGCCTGCAGGAATTGT

TGATAATATTTAGCTCAATAATAATAATAATAATAATAATTCCGTCGAAAAAAAAAAAAA

Supplementary Figure S3: Full-length cDNA of *ApNAC21 22*. The full-length cDNA sequence (996 bp) contains 178 bp 5′ UTR (Yellow highlighted upstream region), 47 bp 3′UTR (Yellow highlighted downstream region) and 771 bp CDS region. Start codon and stop codon have been highlighted with green and red colour, respectively.

CTCCCCCGATACCACCGACCTTCACTCACTCACTTCTCCCCAACCCTACCATGGCCGCCGCCGGAGAT
CTCCATCTGCCGCCGGGATTTCGCTTCCACCCTACCGACGAGGAGCTCGTCATGCACTACCTCTGCCG
GAAATGCTCTGCGCAGTCGATTGCCGTTCCGATTGTGGCCGAAATCGACCTCTACAAGTACGATCCG
TGGGAACTTCCAGGTTTGGCTTTGTACGGCGAGAAAGAGTGGTATTTCTTCTCTCCGAGAGACCGGA
AGTACCCTAACGGTTCGAGACCTAACCGCGCCGCCGGTAGCGGTTACTGGAAGGCCACCGGAGCTG
ATAAACCGATCGGACATCCGAAGCCTGTTGGTATTAAGAAAGCTTTGGTGTTTTACTCCGGCAAGGC
GCCGAAAGGCGAAAAGACGAATTGGATTATGCACGAGTATCGCCTCGCCGACGTCGACCGCTCCGC
CCGCCGGAAGGACCGCAGCCTCAGGCTGGATGAGTGGGTGCTTTGCCGGATATACAACAAGAAGG
GATCAATCGAGAAGCCGCCGGCGACGGCGCCGCCGCAGAGGGAAGCGACGCCGGCGGAGGAAAC
AAAGCCAGCCGTGATGAAGACGGTGGCCGACGCGTCGCCGATGGTGTACGACGAGTTTATGTATCT
TGACCCGTCGGAATCCATGGGGTCCGAGCACGTGCTGTCGCCGGAGGTGGAGAGCACGCCGAAGC
TGACGGAGTGGGAGAAATCCACCCTCGGCTATCCATTCAACTACCTCGGCGGCGGGCCGCCATTGTC
GGCGGCGGCGGCGGCGGCGCAGTTTCAGGGTGGTAACTTTCCGGTGGAGGCGGCAATGCCGGATT
TGTTAGCCTTTATTACAAAGCAGTGCTGAGGTGAACCAATGAGGGGCGTCCACGTGGCATAGGAAA
CTTGCAAAATGACAATTCTATGAAAGTGCCCCCCCCGGAGGAATATGAATGAAAAATTTGTGGATTT
GTAAAAAAAAAAAAAAA

Supplementary Figure S4: Full-length cDNA of *ApNAC02*. The full-length cDNA sequence (1011 bp) contains 50 bp 5′ UTR (Yellow highlighted upstream region), 121 bp 3′UTR (Yellow highlighted downstream region) and 840 bp CDS region. Start codon and stop codon have been highlighted with green and red colour, respectively.

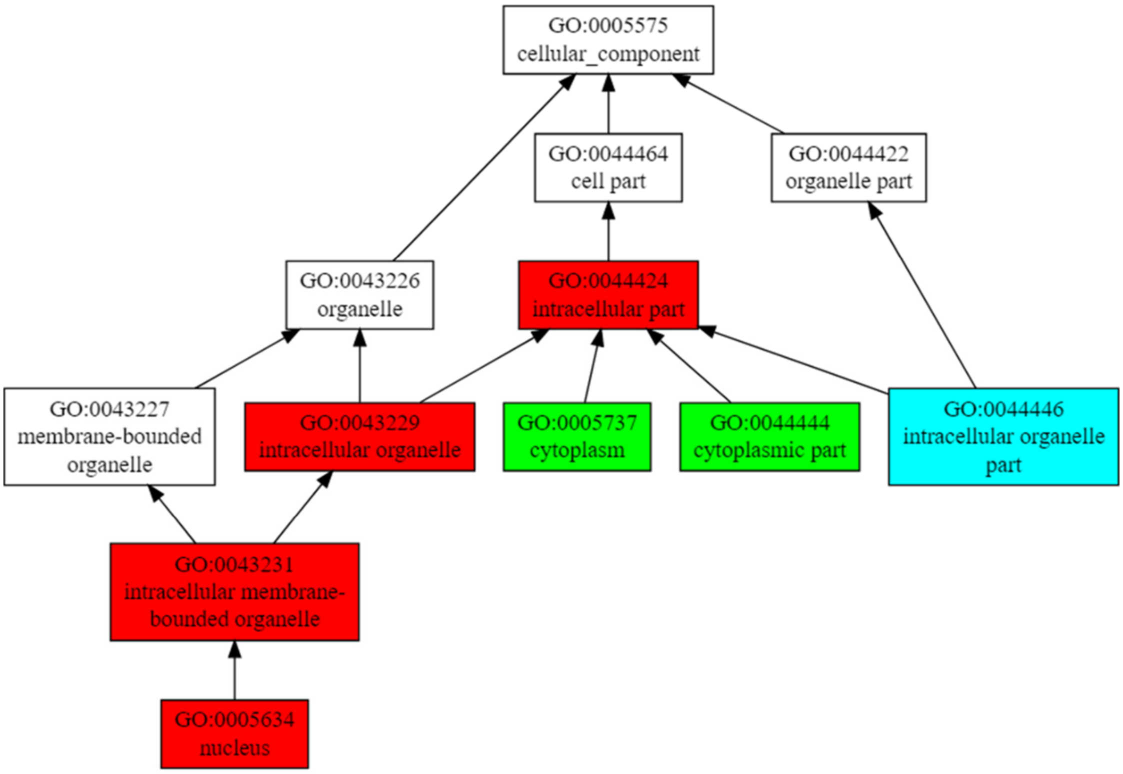

Supplementary Figure S5: Cellular component prediction of *ApNAC83* protein.

LOCTREE 3

Protein Subcellular Localization Prediction System

Protein ID	Score	Expected Accuracy	Localization Class	Gene Ontology Terms	Annotation Type
ApNAC83	48	89%	nucleus	nucleus GO:0005634(IDA);	PSI-BLAST

Predicted Localization: nucleus

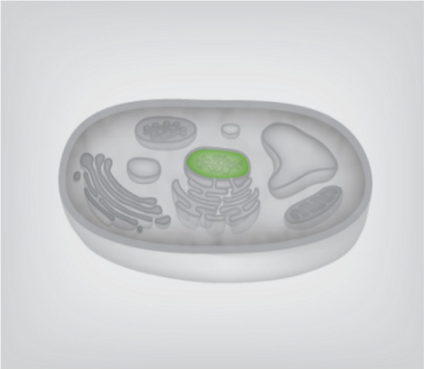

```
>NAC29_ARATH

 Identities = 92/163 (56%), Positives = 110/163 (67%), Gaps = 5/163 (3%)

Query: 12  VLRLPPGFRFHPTDEELVVQYLKRKVASYPLPASVISEVEVCKFDPWDLPGGSE---QEK 68
              LPPGFRFHPTDEEL+V YL+ +  S P P S+I EV++ KFDPW LP  +E    E
Sbjct: 6   QSTLPPGFRFHPTDEELIVYYLRNQTMSKPCPVSIIPEVDIYKFDPWQLPEKTEFGENEW 65

Query: 69  YFFSTREVKYPNGNRSNRATGSGYWKATGLDKQIMSSRSHQIVGMKKTLVFYRGKPPKGC 128
            YFFS RE KYPNG R NRA  SGYWKATG DK I S  S+  VG+KK LVFY+G+PPKG
Sbjct: 66  YFFSPRERKYPNGVRPNRAAVSGYWKATGTDKAIHSGSSN--VGVKKALVFYKGRPPKGI 123

Query: 129 RTDWVMHEYRLSSNSSQQQQLGKDLGQENWVLCRIFLKRRGKK 171
            +TDW+MHEYRL  +     +    + + WVLCRI+ KR   K
Sbjct: 124 KTDWIMHEYRLHDSRKASTKRNGSMRLDEWVLCRIYKKRGASK 166
```

Supplementary Figure S6: Subcellular localization of *ApNAC83*

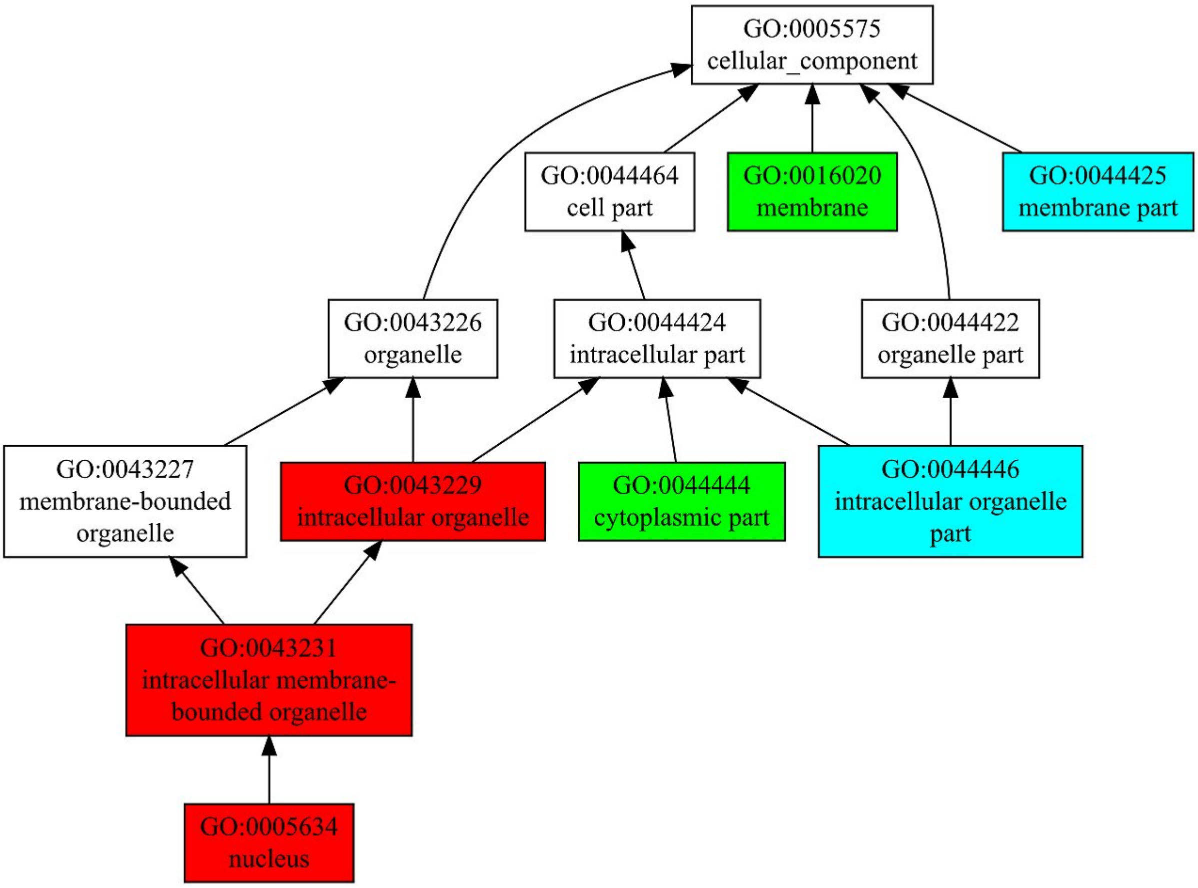

Supplementary Figure S7: Cellular component prediction of *ApNAC21 22* protein.

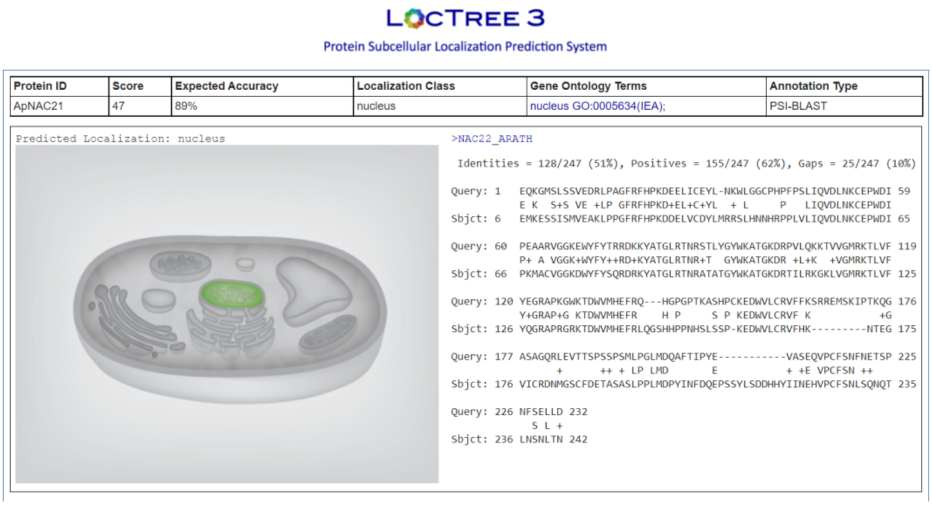

Supplementary Figure S8: Subcellular localization of *ApNAC21 22*.

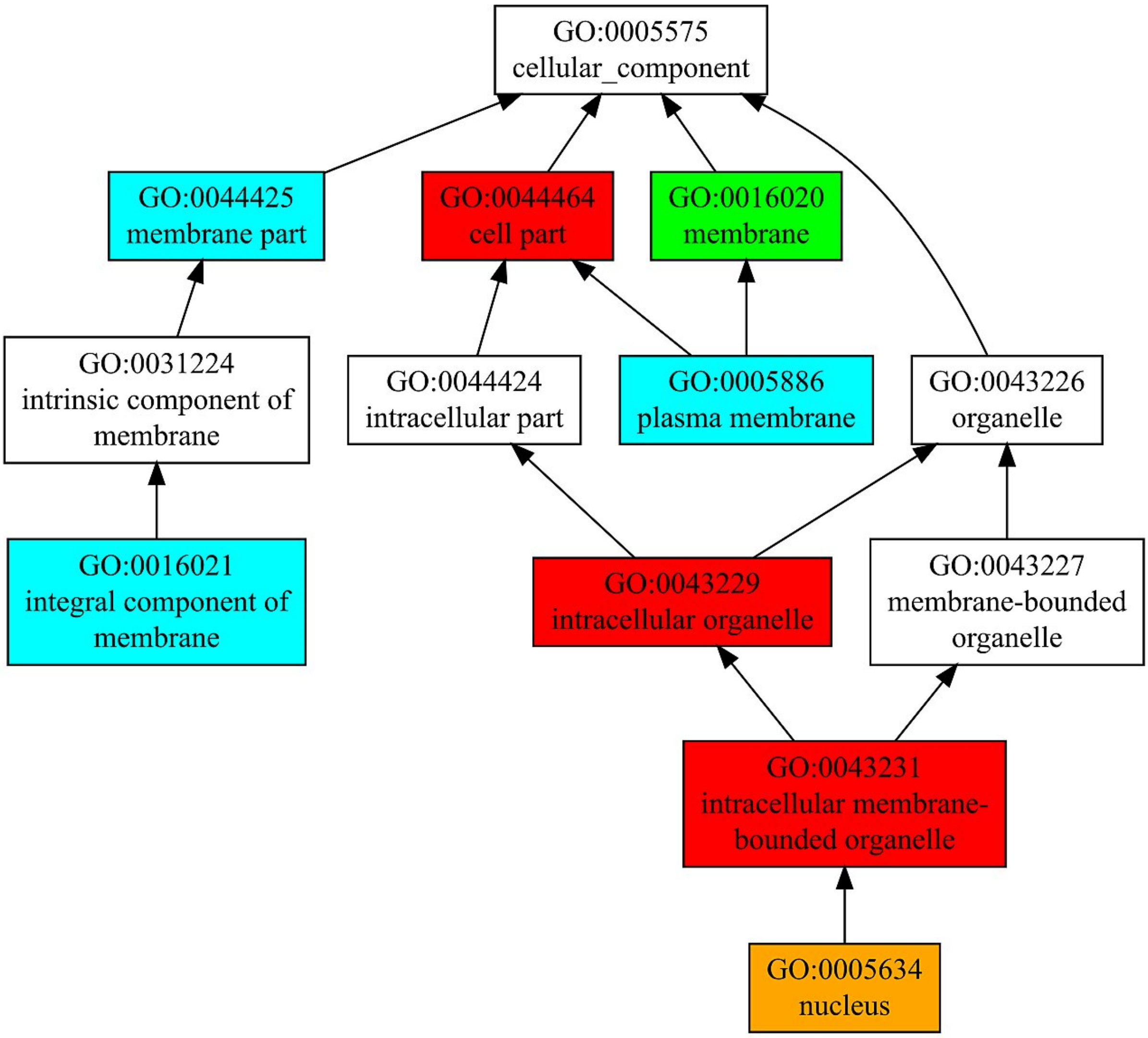

Supplementary Figure S9: Cellular component prediction of *ApNAC02* protein

LOCTREE 3

Protein Subcellular Localization Prediction System

Protein ID	Score	Expected Accuracy	Localization Class	Gene Ontology Terms	Annotation Type
ApNAC02	48	89%	nucleus	nucleus GO:0005634(IDA);	PSI-BLAST

Predicted Localization: nucleus

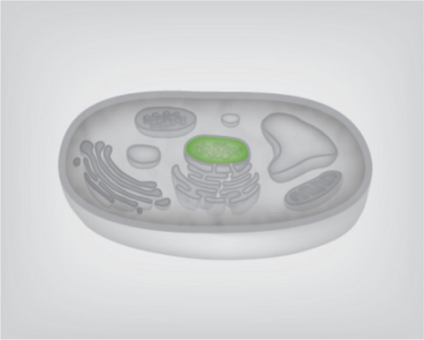

```
>NAC29_ARATH

 Identities = 21/36 (58%), Positives = 30/36 (83%)

Query: 68  MHEYRLADVDRSARRKDRSLRLDEWVLCRIYNKKGS 103
            MHEYRL D  +++ +++ S+RLDEWVLCRIY K+G+
Sbjct: 129 MHEYRLHDSRKASTKRNGSMRLDEWVLCRIYKKRGA 164
```

Supplementary Figure S10: Sub cellular localization of *ApNAC02*

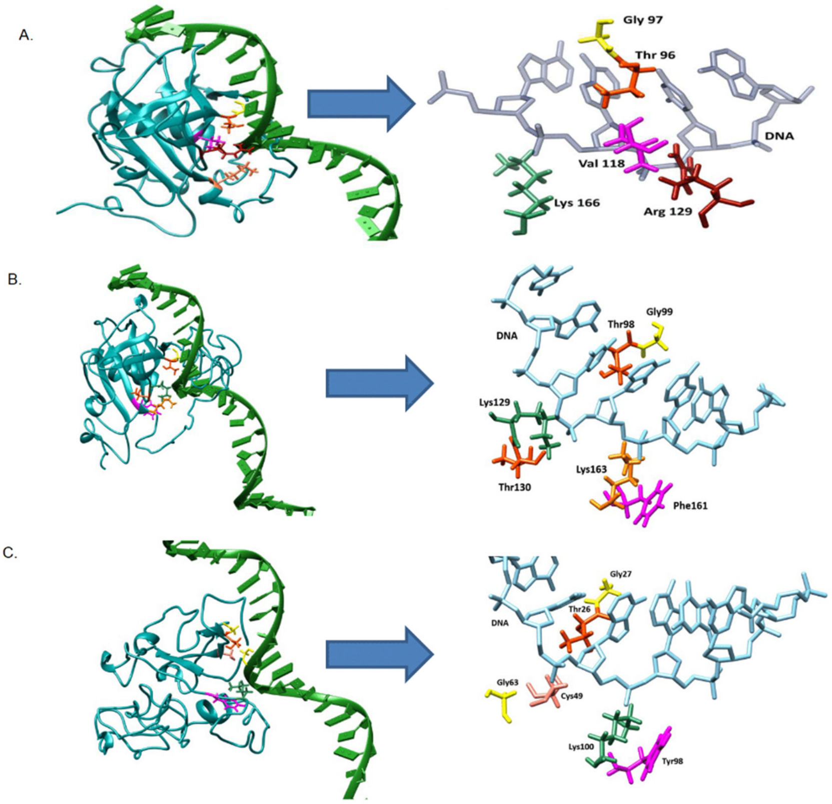

Supplementary Figure S11: DNA binding site prediction: A. predicted structure of *ApNAC83* with DNA. B. predicted the structure of *ApNAC21 22* with DNA. C. predicted structure of *ApNAC02* with DNA

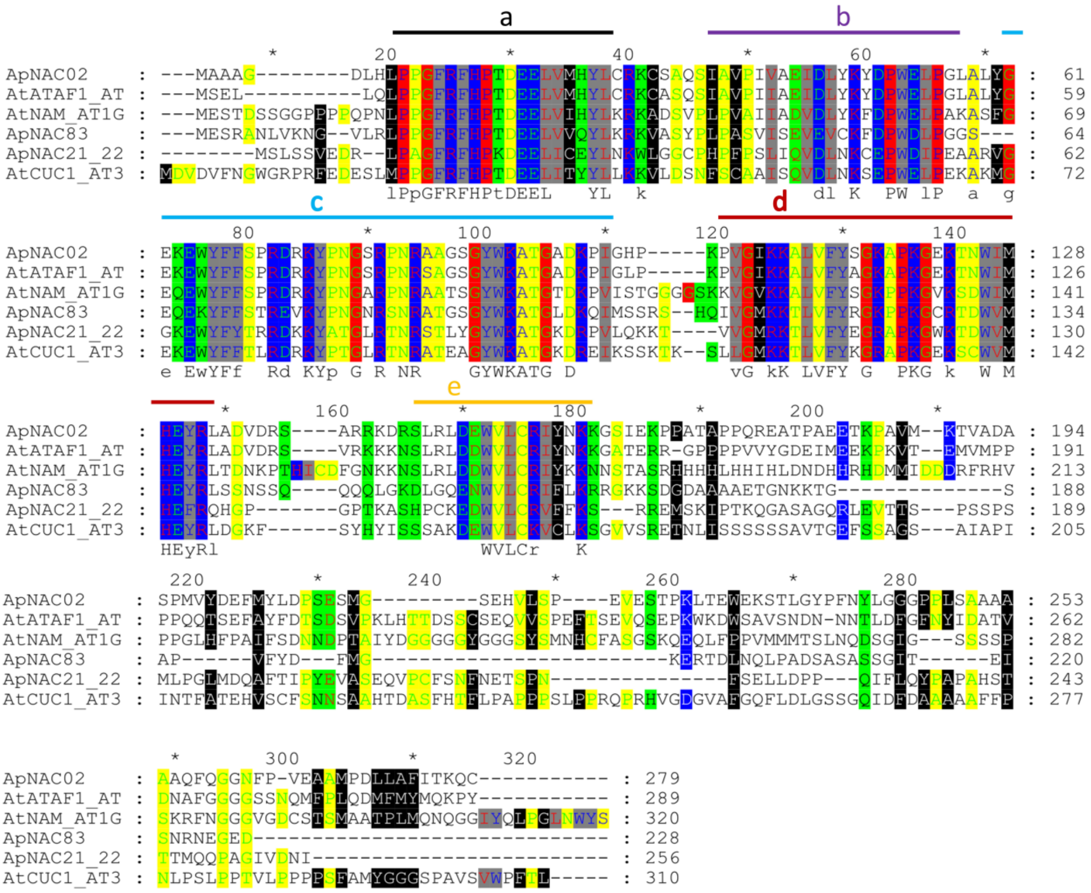

Supplementary Figure S12: Multiple sequence alignment of the deduced amino acid sequences of isolated NACs (*ApNAC02*, *ApNAC21 22*, and *ApNAC83*) along with other known NAC domain proteins from *Arabidopsis thaliana* (*AtNAM*, *AtATAF1*, and *AtCUC1*). The alignment highlighted the positions of the five highly conserved subdomains (a–e) within the NAC domain, visually represented by lines above the sequences.

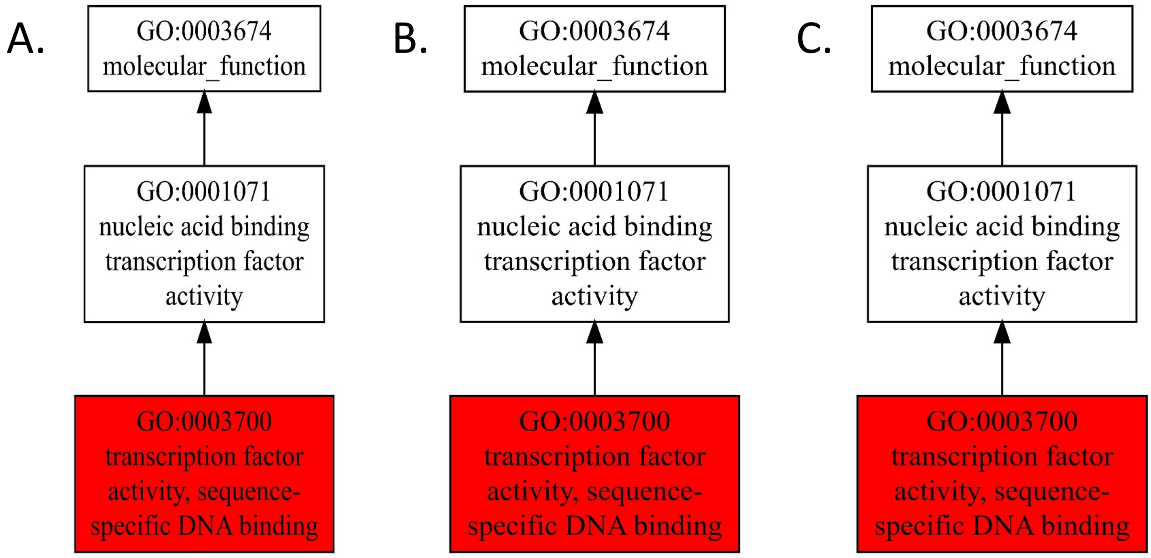

Supplementary Figure S13: Molecular function prediction: A. *ApNAC83*. B. *ApNAC21 22*. C. *ApNAC02*.

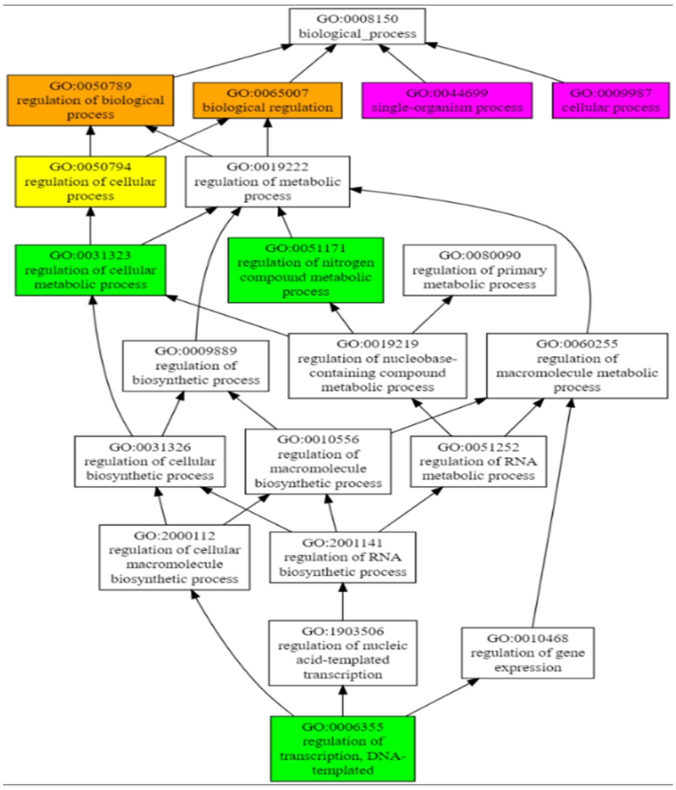

Supplementary Figure S14: Biological process prediction of *ApNAC83*.

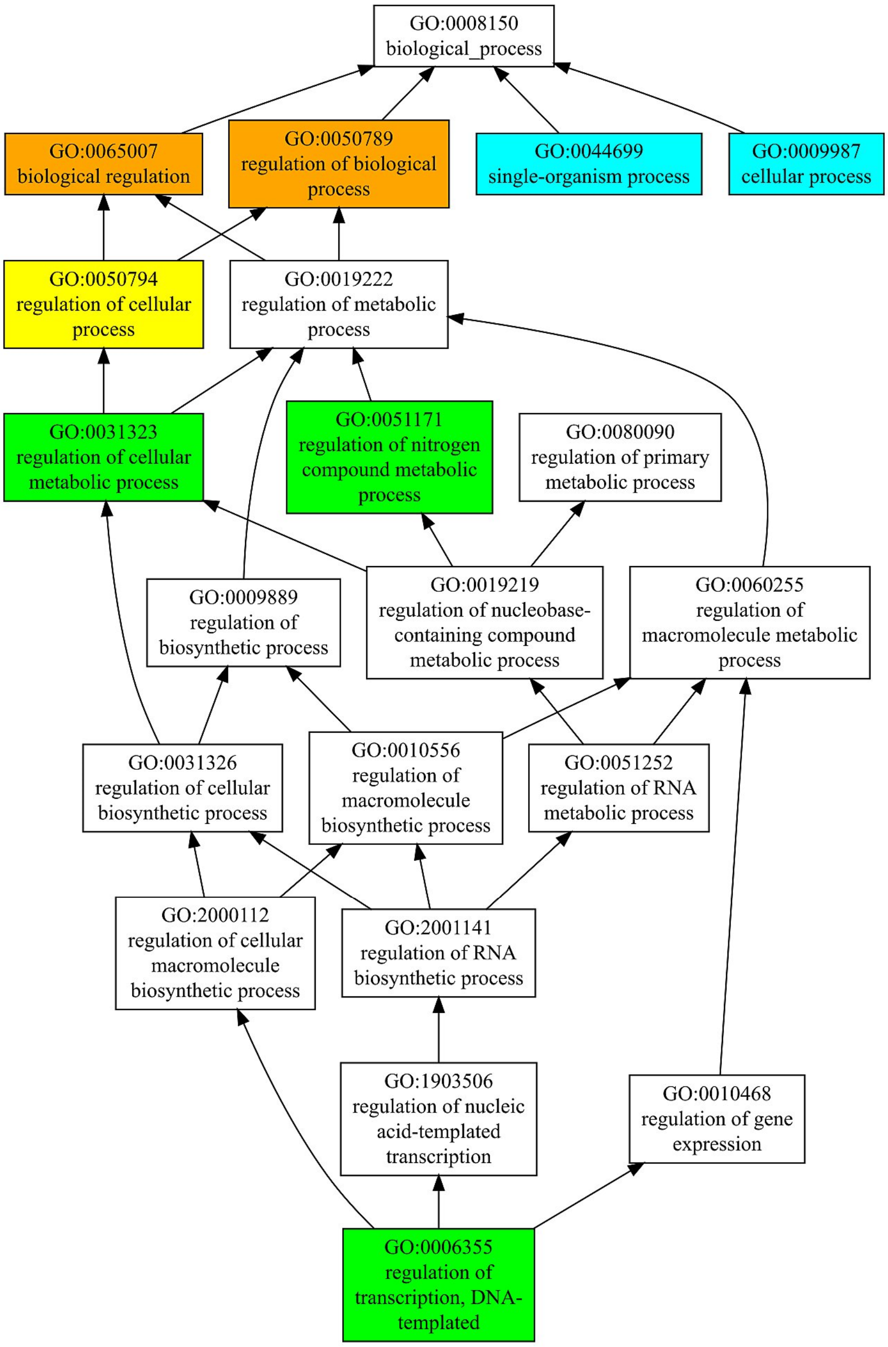

Supplementary Figure S15: Biological process prediction of *ApNAC21 22*.

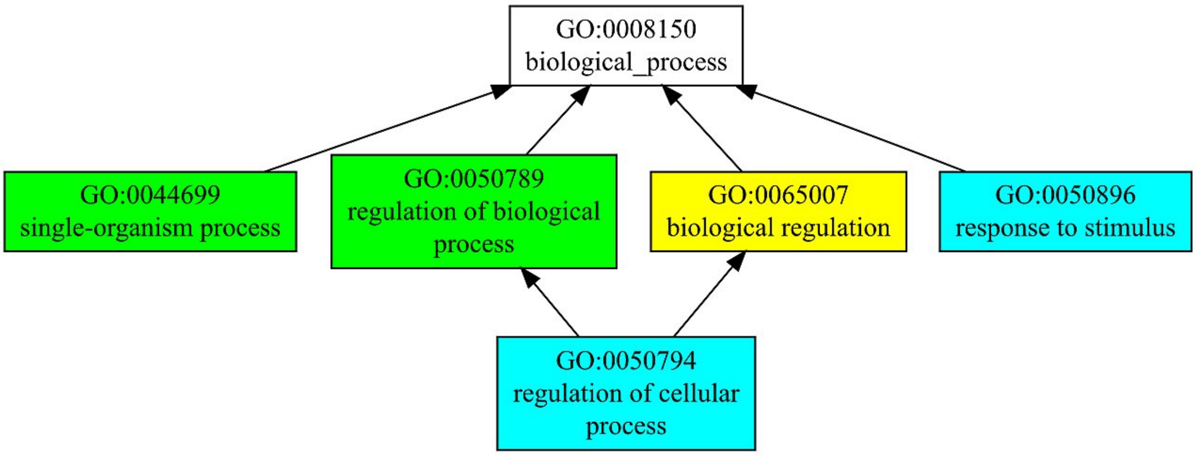

Supplementary Figure S16: Biological process prediction of *ApNAC02.*

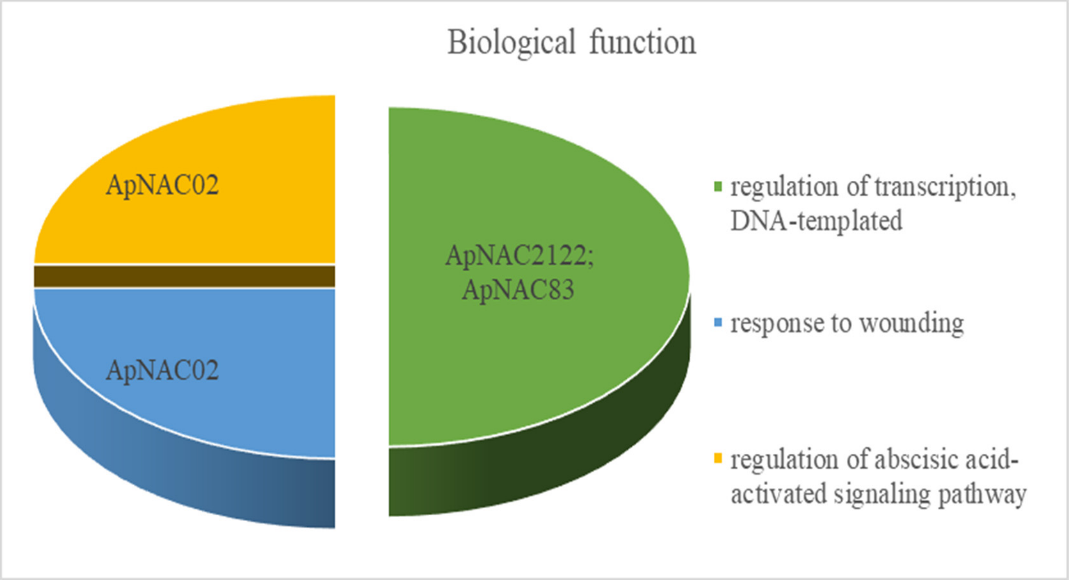

Supplementary Figure S17: Gene ontology classification of NAC gene sequences under biological process.

SHIMADZU LabSolutions

Analysis Report

<Sample Information>

Sample Name	: ABA1		
Sample ID	: ABA1		
Data Filename	: 07-12-21AMIT009.lcd		
Method Filename	: AMIT NM.lcm		
Batch Filename	: amit.lcb		
Vial #	: 1-23	Sample Type	: Unknown
Injection Volume	: 5 uL		
Date Acquired	: 08-12-2021 02:05:56	Acquired by	: System Administrator
Date Processed	: 03-01-2022 18:19:42	Processed by	: System Administrator

<Chromatogram>

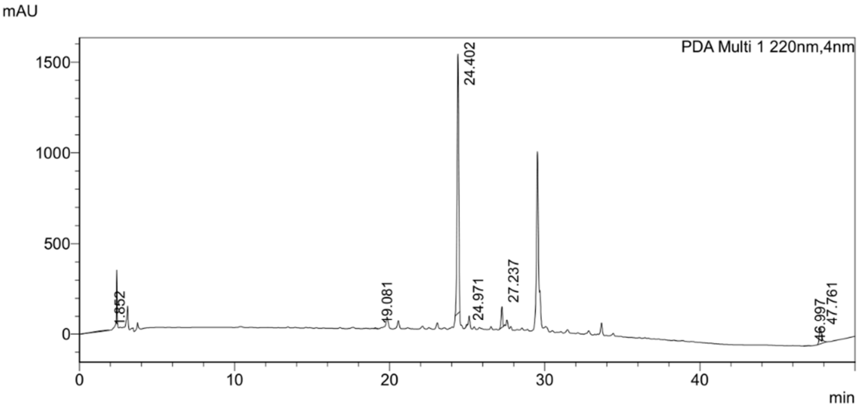

<Peak Table>

PDA Ch1 220nm

Peak#	Ret. Time	Area	Height	Conc.	Name
1	1.852	257608	2519	0.000	
2	19.081	26953	4150	0.018	AP-4
3	24.402	10442144	1427079	1.847	AP-1
4	24.971	41233	7509	0.134	AP-3
5	27.237	738918	117175	0.405	AP-2
6	46.997	19381	1783	0.000	
7	47.761	1023940	85048	0.000	
Total		12550176	1645263		

Supplementary Figure S18: HPLC analysis report of four different bioactive of *Andrographis paniculata* under ABA treatment (0h): where AP-1 is andrographolide and AP-2 is neo-andrographolide (AP-2), AP-3 is 14-deoxyandrographolide, and AP-4 is andrographolide glycoside

SHIMADZU LabSolutions

Analysis Report

<Sample Information>

Sample Name	: ABA2		
Sample ID	: ABA2		
Data Filename	: 07-12-21AMIT010.lcd		
Method Filename	: AMIT NM.lcm		
Batch Filename	: amit.lcb		
Vial #	: 1-24	Sample Type	: Unknown
Injection Volume	: 5 uL		
Date Acquired	: 08-12-2021 02:56:34	Acquired by	: System Administrator
Date Processed	: 03-01-2022 18:19:46	Processed by	: System Administrator

<Chromatogram>

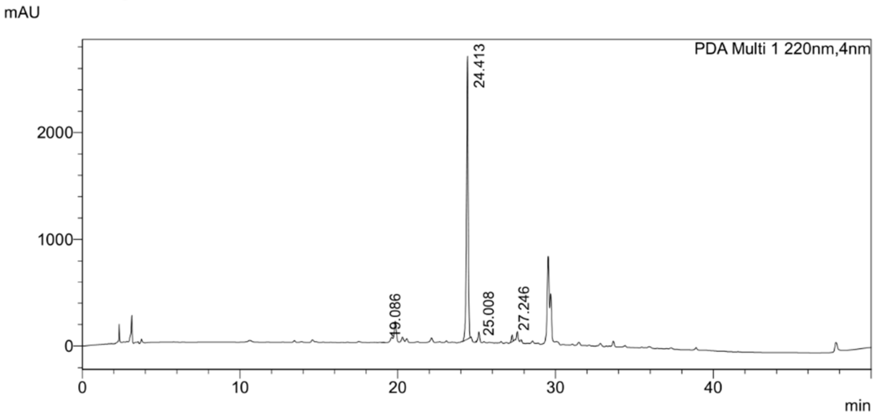

<Peak Table>

PDA Ch1 220nm

Peak#	Ret. Time	Area	Height	Conc.	Name
1	19.086	29522	4564	0.019	AP-4
2	24.413	19296887	2649630	3.406	AP-1
3	25.008	52154	9629	0.163	AP-3
4	27.246	367877	62395	0.204	AP-2
Total		19746440	2726218		

Supplementary Figure S19: HPLC analysis report of four different bioactive of *Andrographis paniculata* under ABA treatment (6h): where AP-1 is andrographolide and AP-2 is neo-andrographolide (AP-2), AP-3 is 14-deoxyandrographolide, and AP-4 is andrographolide glycoside.

SHIMADZU LabSolutions

Analysis Report

<Sample Information>

Sample Name	: ABA3
Sample ID	: ABA3
Data Filename	: 07-12-21AMIT011.lcd
Method Filename	: AMIT NM.lcm
Batch Filename	: amit.lcb
Vial #	: 1-25
Injection Volume	: 5 uL
Date Acquired	: 08-12-2021 03:47:13
Date Processed	: 03-01-2022 18:19:53

Sample Type	: Unknown
Acquired by	: System Administrator
Processed by	: System Administrator

<Chromatogram>

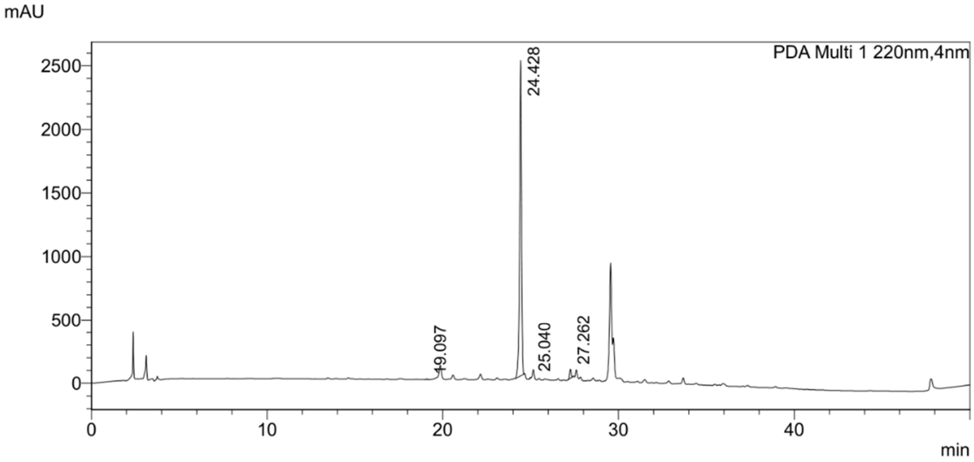

<Peak Table>

PDA Ch1 220nm

Peak#	Ret. Time	Area	Height	Conc.	Name
1	19.097	23685	3497	0.017	AP-4
2	24.428	18387554	2483306	3.246	AP-1
3	25.040	23224	-298	0.085	AP-3
4	27.262	424577	72995	0.234	AP-2
Total		18859041	2559500		

Supplementary Figure S20: HPLC analysis report of four different bioactive of *Andrographis paniculata* under ABA treatment (12h): where AP-1 is andrographolide and AP-2 is neo-andrographolide (AP-2), AP-3 is 14-deoxyandrographolide, and AP-4 is andrographolide glycoside.

SHIMADZU LabSolutions **Analysis Report**

<Sample Information>

Sample Name : ABA4
Sample ID : ABA4
Data Filename : 07-12-21AMIT012.lcd
Method Filename : AMIT NM.lcm
Batch Filename : amit.lcb
Vial # : 1-26
Injection Volume : 5 uL
Date Acquired : 08-12-2021 04:37:52
Date Processed : 03-01-2022 18:19:57

Sample Type : Unknown
Acquired by : System Administrator
Processed by : System Administrator

<Chromatogram>

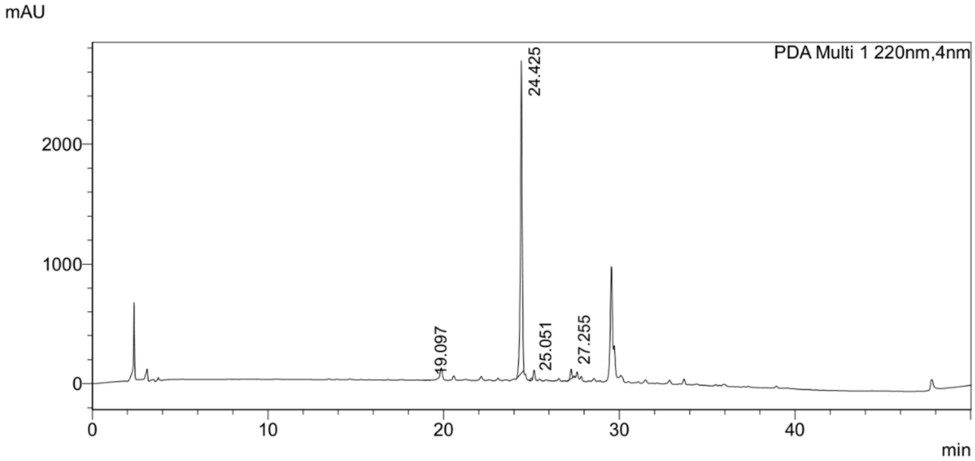

<Peak Table>

PDA Ch1 220nm

Peak#	Ret. Time	Area	Height	Conc.	Name
1	19.097	23972	3461	0.017	AP-4
2	24.425	18679647	2608079	3.297	AP-1
3	25.051	148528	18344	0.422	AP-3
4	27.255	458682	81431	0.253	AP-2
Total		19310829	2711315		

Supplementary Figure S21: HPLC analysis report of four different bioactive of *Andrographis paniculata* under ABA treatment (24h): where AP-1 is andrographolide and AP-2 is neo-andrographolide (AP-2), AP-3 is 14-deoxyandrographolide, and AP-4 is andrographolide glycoside.

SHIMADZU LabSolutions

Analysis Report

<Sample Information>

Sample Name	: STD MIX		
Sample ID	: CAL6		
Data Filename	: 07-12-21AMIT006.lcd		
Method Filename	: AMIT NM.lcm		
Batch Filename	: amit.lcb		
Vial #	: 1-29	Sample Type	: Standard
Injection Volume	: 4 uL	Level	: 6
Date Acquired	: 07-12-2021 23:33:59	Acquired by	: System Administrator
Date Processed	: 03-01-2022 18:21:30	Processed by	: System Administrator

<Chromatogram>

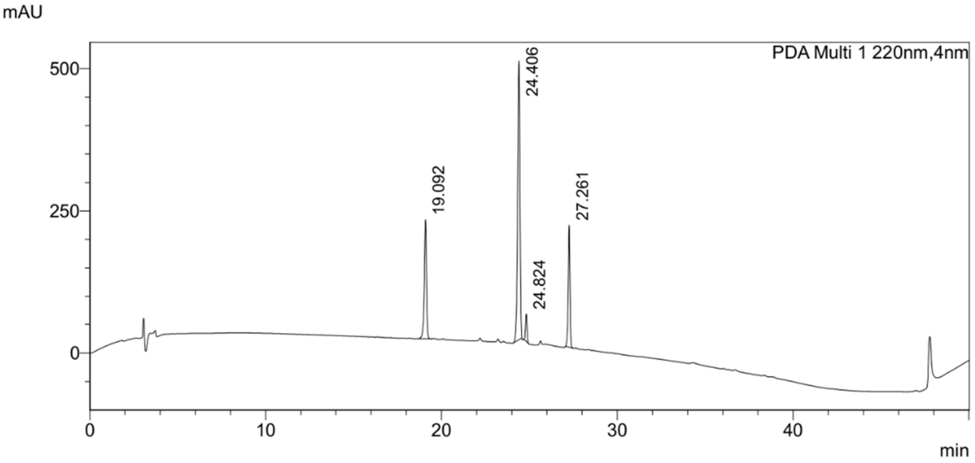

<Peak Table>

PDA Ch1 220nm

Peak#	Ret. Time	Area	Height	Conc.	Name
1	19.092	1804867	209594	0.801	AP-4
2	24.406	4493540	490568	0.799	AP-1
3	24.824	291446	48994	0.805	AP-3
4	27.261	1455300	215327	0.794	AP-2
Total		8045152	964483		

Supplementary Figure S22: HPLC analysis report of four different standards from *Andrographis paniculata* used for quantification. where AP-1 is andrographolide and AP-2 is neo-andrographolide (AP-2), AP-3 is 14-deoxyandrographolide, and AP-4 is andrographolide glycoside.

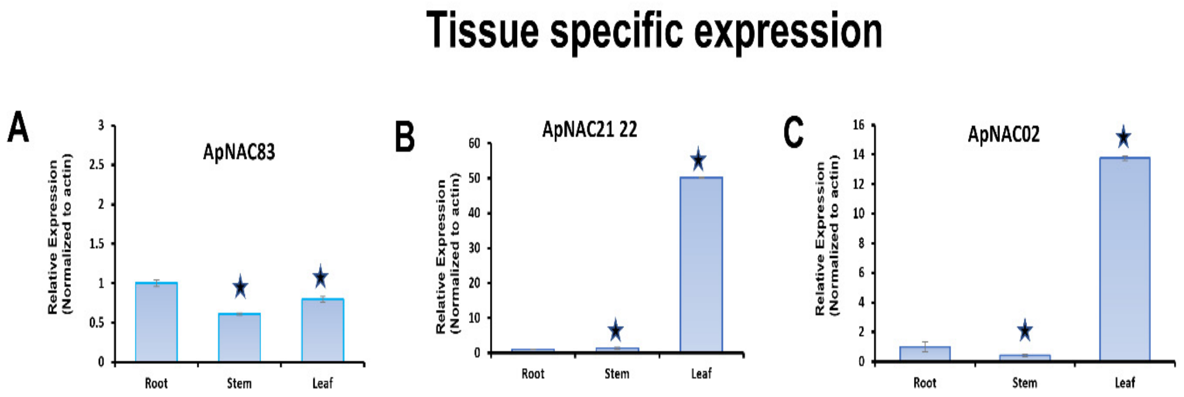

Supplementary Figure S23: Tissue-specific (root, stem, and leaves) relative expression of isolated genes: (A) *ApNAC83* (B) *ApNAC21 22*; (C) *ApNAC02*. Differences were scored as statistical significance at *P < 0.05. Asterisk symbols indicate significance.

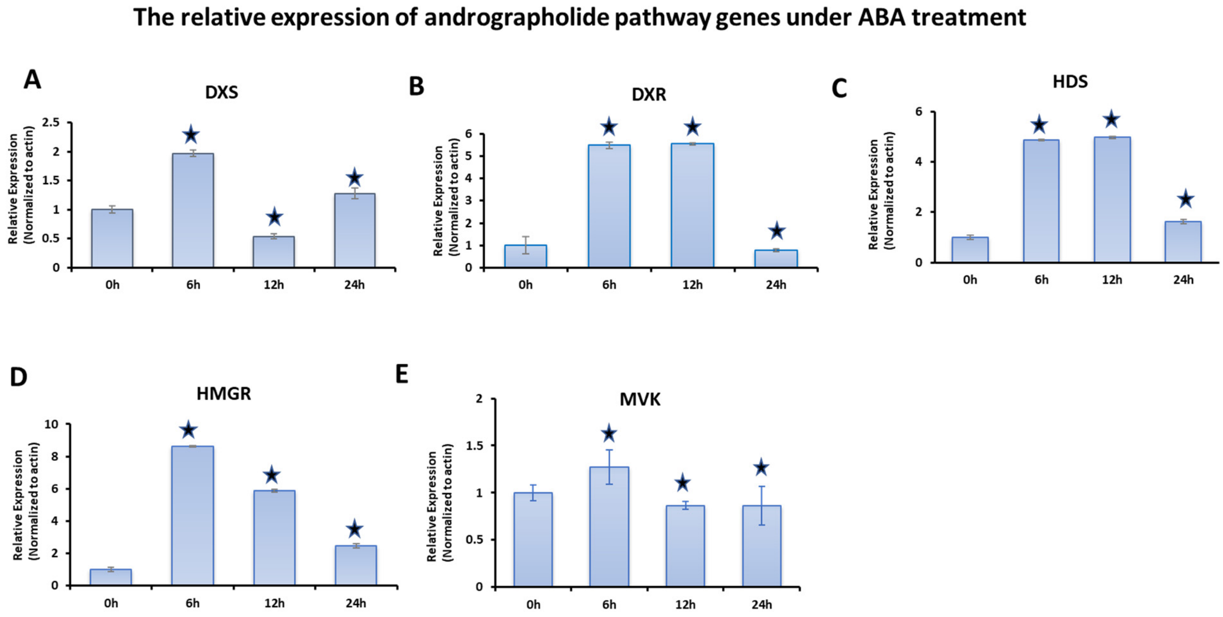

Supplementary Figure S24: Relative expression of andrographolide pathway related genes in response to ABA treatment at a different time interval (0h, 6h, 12h, and 24h). Differences were scored as statistical significance at *P < 0.05. Asterisk symbols indicate significance.

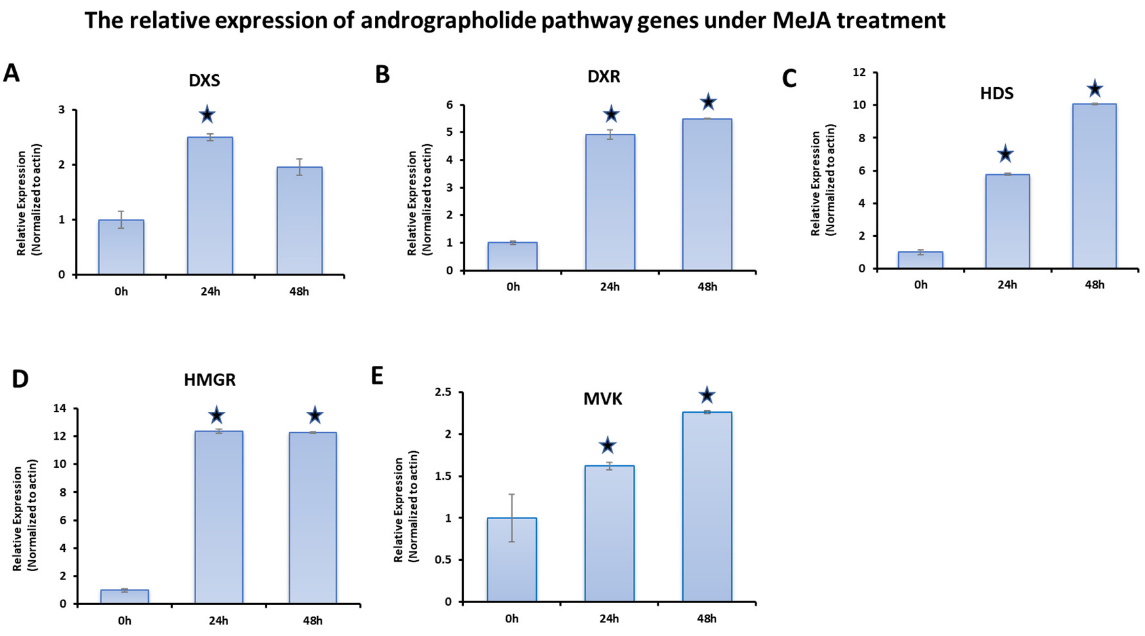

Supplementary Figure S25: Relative expression of andrographolide pathway related genes in response to MeJA treatment at a different time interval (0h, 24h, and 48h). Differences were scored as statistical significance at *P < 0.05. Asterisk symbols indicate significance.
